# miRNA gene mutations commonly disrupt the proper functioning of miRNA genes

**DOI:** 10.1126/sciadv.aea6079

**Published:** 2026-02-18

**Authors:** Magdalena Machowska, Natalia Szostak, Adrian Tire, Wladyslaw Wegorek, Malwina Suszynska, Arkadiusz Kajdasz, Paulina Galka-Marciniak, Anna Philips, Piotr Kozlowski

**Affiliations:** ^1^ Institute of Bioorganic Chemistry, Polish Academy of Sciences, Poznan, Poland.; ^2^ Laboratory of Nuclear Proteins, Faculty of Biotechnology, University of Wroclaw, Wroclaw, Poland.

## Abstract

A growing number of mutations are being identified in the noncoding genome, including microRNA (miRNA) genes; however, little is known about the consequences of these mutations and how harmful they are to the functioning of miRNA genes. To evaluate the effects of miRNA gene mutations, we took advantage of a large collection of somatic mutations identified in miRNA genes in >10,000 The Cancer Genome Atlas cancer samples and compared them with the corresponding miRNA sequencing data. Using different analytical approaches and rigorous statistical criteria, we revealed that a substantial fraction of mutations is deleterious for the proper functioning of miRNA genes affecting the level of mature miRNAs, isomiR profiles (precision of DROSHA/DICER1 cleavage), and/or 5p/3p miRNA strand balance. We also showed that most mutations, especially those identified as deleterious, destabilize the structure of miRNA precursors. The analysis showed that many miRNA gene mutations can damage miRNA genes and, if located in disease-related miRNA genes, may be pathogenic variants.

## Teaser

Most mutations located in miRNA genes seriously affect their proper functioning.

## INTRODUCTION

MicroRNAs (miRNAs) are small [~20 to 23 nucleotides (nt)] noncoding regulatory RNAs that are estimated to modulate the expression of almost all human genes (*1*). Over three decades of investigations have shown that miRNAs play important roles in the regulation of many cellular and physiological processes (*2*, *3*), and many miRNAs have been found to be up-regulated or down-regulated in specific physiological conditions or diseases (*4*). The two best-known databases of miRNAs, miRBase (*5*) and MirGeneDB (*6*), cumulatively annotate ~2000 miRNAs/miRNA genes in humans, including ~600 of high confidence.

Although the genetic variation in the noncoding parts of the genome is still largely understudied, an increasing number of genetic variants have been identified in miRNA genes, including common and rare single-nucleotide polymorphisms (SNPs), sporadic germline mutations, and cancer somatic mutations (*7*–*10*). Progress in the identification of noncoding variants in the noncoding genome has been facilitated by the increased popularity of whole-genome sequencing.

However, although there are still few functional analyses of mutations in miRNA genes, those performed have focused almost exclusively on mutations located in the seeds of mature miRNAs, which constitute only a small fraction of miRNA gene/precursor sequences. A few such mutations have been proven to be causative variants of rare Mendelian diseases (germline mutations) (*9*, *11*–*14*) or to affect the functionality of miRNA genes in cancer (somatic mutations) (*15*–*19*). On the other hand, almost nothing is known about the consequences of mutations located in other parts of miRNA genes, especially in their most crucial parts, i.e., the sequence encoding the precursor miRNA (pre-miRNA) hairpin and its immediate flanking sequences. To the best of our knowledge, the only study that has attempted systematic analysis of the genetic variants in miRNA genes was performed 15 years ago (in the pre–next-generation sequencing era). Rossi’s team (*20*), with the use of simple in vitro molecular tests (luciferase assays and northern blots), analyzed a few SNPs identified at that time in different parts of miRNA genes. The results of this study suggested that most of the genetic variants in miRNA genes affect miRNA biogenesis and/or function. Because of the lack of information on the consequences of genetic variants in miRNA genes, distinguishing neutral variants from destructive variants (deleterious to miRNA genes and likely functional) is difficult. This distinction between likely neutral (synonymous) and likely functional or deleterious (missense, nonsense, and frameshift splice-site) variants in protein-coding genes greatly facilitates many aspects of research on these genes, including prioritizing variants for further analysis.

Canonical miRNAs are generated in a multistep process (*21*–*23*), the most important of which is the stepwise processing of long primary miRNA precursors by the nucleases DROSHA and DICER1. Each step of miRNA biogenesis and processing strongly depends on different structural and/or sequence motifs (*24*, *25*). The alterations in these motifs via genetic variants/mutations, not only those located in seed regions, may lead to erroneous functioning of miRNA genes, including differences in the efficiency of mature miRNA release and generation of altered miRNAs. The variants may act as riboSNitches, affecting the structure of the precursors (*26*), altering the thermodynamic properties of the precursors, and/or directly affecting key functional elements of miRNA precursors, such as DROSHA/DICER1 cleavage sites or the binding motifs of regulatory proteins. Depending on the effect of the variant, it may be considered a loss- or gain-of-function mutation. The further arguments that may support the potential functional/deleterious effect of mutations in miRNA genes are (i) the decreased frequency of SNPs (purifying selection) in these regions (*27*, *28*); (ii) the results of testing artificial miRNA precursors/short hairpin RNAs, in which the tested sequence changes often resulted in altered processing of the precursors (*29*–*32*); and (iii) the results demonstrating the sequence specificity of DROSHA or DICER1 cleavage sites (*33*–*35*).

To shed more light on the consequences of mutations in miRNA genes (defined as sequences encoding pre-miRNAs with immediately adjacent 25-nt flanking sequences, roughly corresponding/overlapping with miRNA genes designated by the HUGO Gene Nomenclature Committee) on miRNA biogenesis, we took the advantage of a large collection (*n* ~ 7000) of somatic mutations identified and annotated in miRNA genes (*7*) in cancer samples from The Cancer Genome Atlas (TCGA) project and the corresponding miRNA sequencing data annotated with the use of isoMiRmap (*36*). IsoMiRmap is a recently developed tool that allows precise annotation of miRNA reads considering length (isomiRs) and sequence (mutations) variation (*36*). The comparison of the particular mutations with the corresponding miRNA sequencing data allowed us to evaluate in real samples (not artificial functional models) the effect of the substantial number of mutations on miRNA levels, generated isomiRs, and the proportion of generated miRNA strands (5p/3p strand balance). In addition, with the use of RNA sequencing (RNA-seq) data for the selected mutations, we evaluated the effects of the mutations on the levels of the target mRNAs. The performed analyses revealed that a substantial number of tested mutations severely affect the function of miRNA genes and allow the identification of mutations with the most notable effects on particular aspects of biogenesis. Still, the fraction of mutations detected as functional is likely severely underestimated because of the limited power of some comparisons, resulting from the inherent characteristics of cancer samples, such as contamination with normal tissue, heterogeneity, and the low allelic frequency of some mutations. Our results are consistent with the notion that miRNA precursors are rather fragile structures in which even subtle changes affect their processing and suggest that most mutations in miRNA genes should be considered likely functional (deleterious or gain-of-function) rather than neutral variants.

## RESULTS

### Data processing

**Fig. 1.**
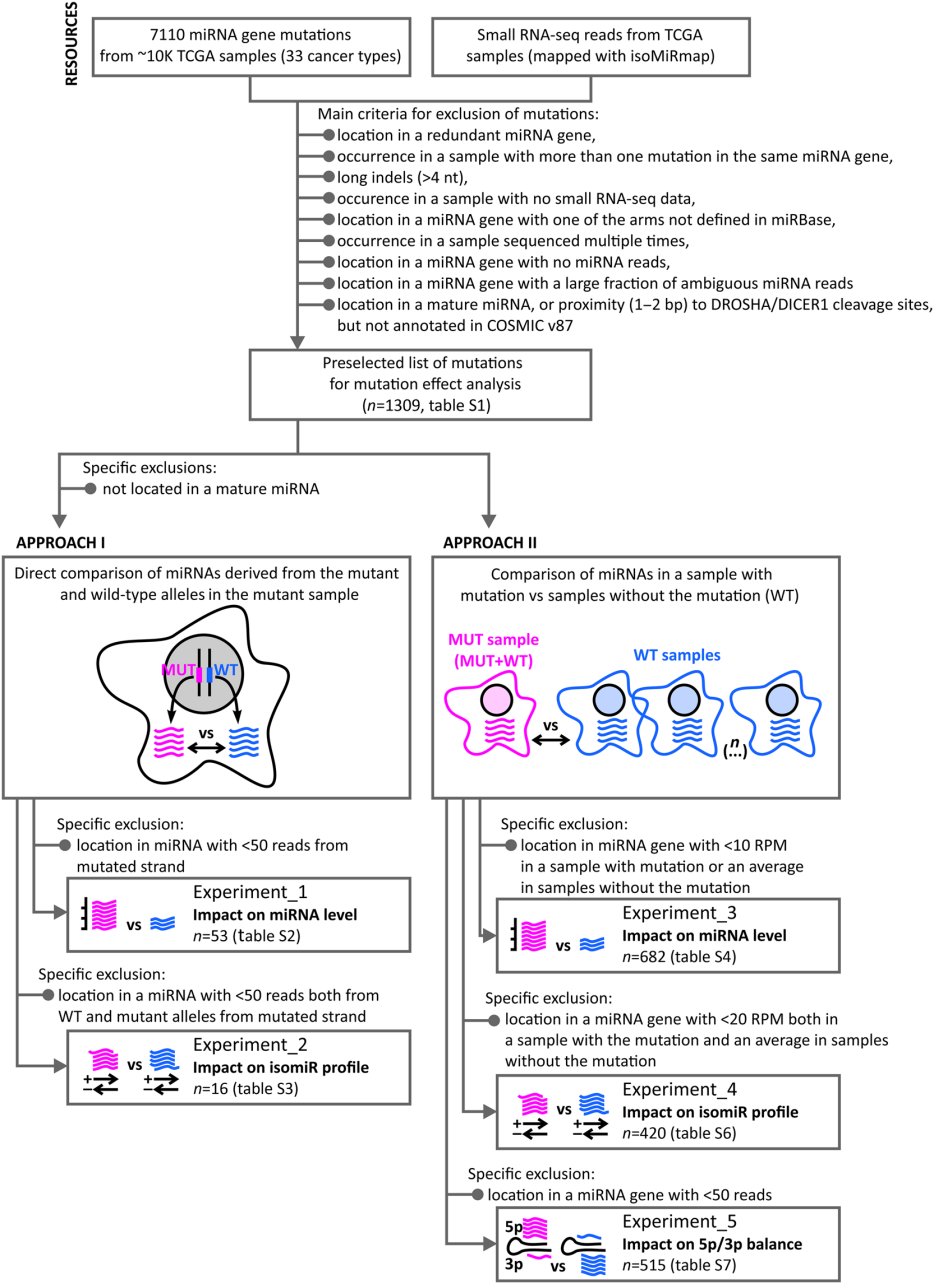
Workflow scheme and mutation selection criteria for the analysis. MUT, mutant; WT, wild type.

To evaluate the effects of mutations in miRNA genes on the levels and sequences of generated miRNAs, we compiled data from the list of 7110 cancer somatic mutations identified in miRNA genes in >10,000 TCGA cancer samples representing 33 cancer types (*7*) and from the corresponding miRNA reads [small RNA-seq (sRNA-seq) datasets] mapped using isoMiRmap (for details, see Materials and Methods) (*36*). To avoid ambiguous and/or false-positive results in subsequent analyses, different inclusion/exclusion criteria were applied to the analyzed mutations and samples, as described in Materials and Methods and presented in Fig. 1. The remaining 1309 mutations listed and characterized in table S1 served as a resource of mutations for subsequent analyses. The effect of mutations on miRNAs expressed from the mutated genes was analyzed using two general approaches, i.e., comparing miRNAs expressed from a particular miRNA gene from the mutant (MUT) and wild-type (WT) alleles in the mutated sample (APPROACH_1) and comparing miRNAs expressed from a mutated gene in the sample with the mutation with other samples of the same cancer type without the mutation (APPROACH_2). The general concept of the study, as well as the exclusion criteria and the number of mutations selected for subsequent experiments, is shown in Fig. 1 and fig. S1.

### Analysis of the effect of miRNA gene mutations by a direct comparison of miRNAs generated from mutated and wild-type alleles in individual samples with mutations

In this approach, we took advantage of the fact that some mutations may be directly observed at the RNA level; therefore, direct observation of the miRNAs derived from the mutant and wild-type alleles in one sample is possible without biases resulting from the biological and technical differences between samples or their processing/analysis. The limitation of such an approach, however, is that it allows the analysis only of mutations located in sequences encoding mature miRNAs (expressed at the miRNA level).

**Fig. 2.**
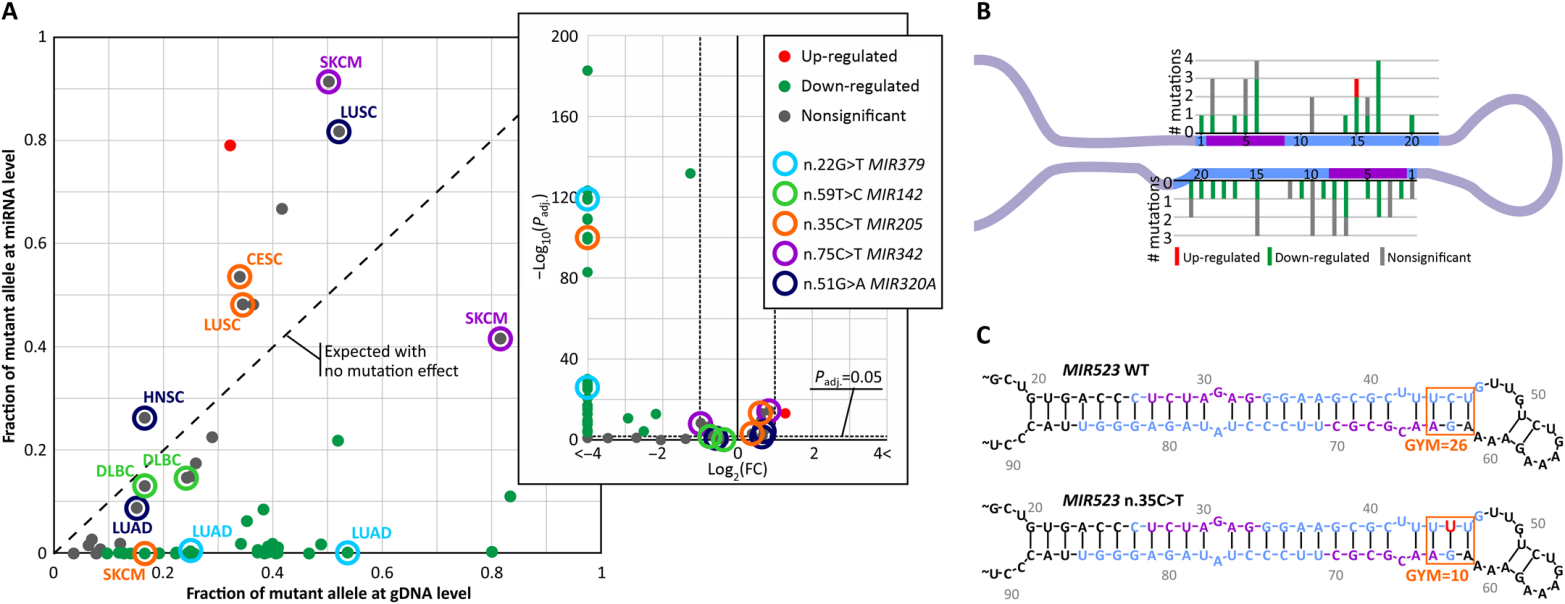
Effects of miRNA gene mutations on miRNA levels (Experiment_1). (**A**) Comparison of the proportion of MUT reads at the DNA and miRNA levels; inset: volcano plot showing log_2_(FC) values of the fraction of mutated miRNAs compared with the fraction of mutated allele at the DNA level (*x* axis) and −log_10_(adjusted *P* value) of Fisher’s exact test (*y* axis). Each dot represents one mutation; red, green, and gray dots indicate mutations increasing, decreasing, and not significantly changing the miRNA level, respectively. The color circles indicate mutations of the same type occurring in different samples. gDNA, genomic DNA. (**B**) Distribution of the analyzed mutations along the consensus miRNA precursor structure. (**C**) Structure of the *MIR523* precursor with the GYM motif altered by the n.35C>T mutation.

In the first experiment (Experiment_1), to investigate the effects of mutations on miRNA levels, we compared, at the genomic (DNA) and transcript (mature miRNA) levels, the allelic fractions (proportions of reads with mutations) of 53 mutations fulfilling criteria of analysis. Unexpectedly, as shown in Fig. 2A, there was very little relationship between the fractions of mutant alleles at the DNA and RNA levels, and most mutations (*n* = 32, 60%) significantly (adjusted *P* < 0.05; Fisher’s exact test) deviated from the trendline (*x* = *y*), representing an equal proportion of mutant reads at the DNA and miRNA levels, expected for mutations with no effect on the miRNA level. Only one mutation significantly increased the miRNA level [adjusted *P* < 0.05; absolute fold change (FC) ≥2], whereas the vast majority of these mutations decreased the miRNA level of mutated alleles (1 versus 31; *P* = 0.00003; Fisher’s exact test), in most cases almost to 0. Moreover, the relative decreases had much larger amplitudes [mostly log(FC) < −2] than the relative increases in miRNA levels [log(FC) ~ 1] (Fig. 2A, inset). As shown in Fig. 2B, the mutations affecting miRNA levels are roughly equally distributed along mature miRNA sequences and do not cluster in any specific position of the precursor (details regarding each of the mutations are listed in table S2).

It is noteworthy that several mutations (*n* = 5) that occurred in more than one sample, especially in samples of the same cancer type, generally had consistent effects on miRNA levels (Fig. 2A, inset), further confirming the validity of the analysis. Among these mutations, n.22G>T in *MIR379* was identified in two lung adenocarcinoma (LUAD) samples and, in both cases, acutely decreased the level of mutant miRNA [log_2_(FC) = −6 and −8.5], whereas n.75C>T in *MIR342* [in two skin cutaneous melanoma (SKCM) samples], n.59T>C in *MIR142* [in two lymphoid neoplasm diffuse large B cell lymphoma (DLBC) samples], and n.51G>A in *MIR320A* [in LUAD, lung squamous cell carcinoma (LUSC), and head and neck squamous cell carcinoma (HNSC) samples] had no significant effect on the miRNA level. Only n.35C>T in *MIR205* had a discordant effect on the miRNA level in the SKCM sample [decrease, log_2_(FC) = −7] versus the cervical squamous cell carcinoma and endocervical adenocarcinoma (CESC) and LUSC samples (no significant change). These discrepancies may, however, result from differences in cancer/tissue type, differences in mutation allelic frequency, and general variation in cancer samples.

Recently, it was shown that the nucleotide composition of a 3–base pair–long motif of the miRNA precursor duplex encompassing the DICER1 cleavage site, called GYM, affects the efficiency of DICER1 processing and thus may affect miRNA levels (*37*). By comparing the GYM scores of the three mutations located in the GYM region with those of their wild-type counterparts, we found that the effect of one of the mutations, i.e., n.35C>T in *MIR523*, which decreases the miRNA level [log_2_(FC) = −6.5], may be well explained by the GYM effect, i.e., the GYM score decreased from 26 for the wild-type precursor to 10 for the mutant precursor (the GYM score ranges from 0 to 100, with higher values indicating more efficient processing; Fig. 2C). Notably, however, miRNA levels can be influenced by many other factors, including the structure of the miRNA precursor (discussed below).

**Fig. 3.**
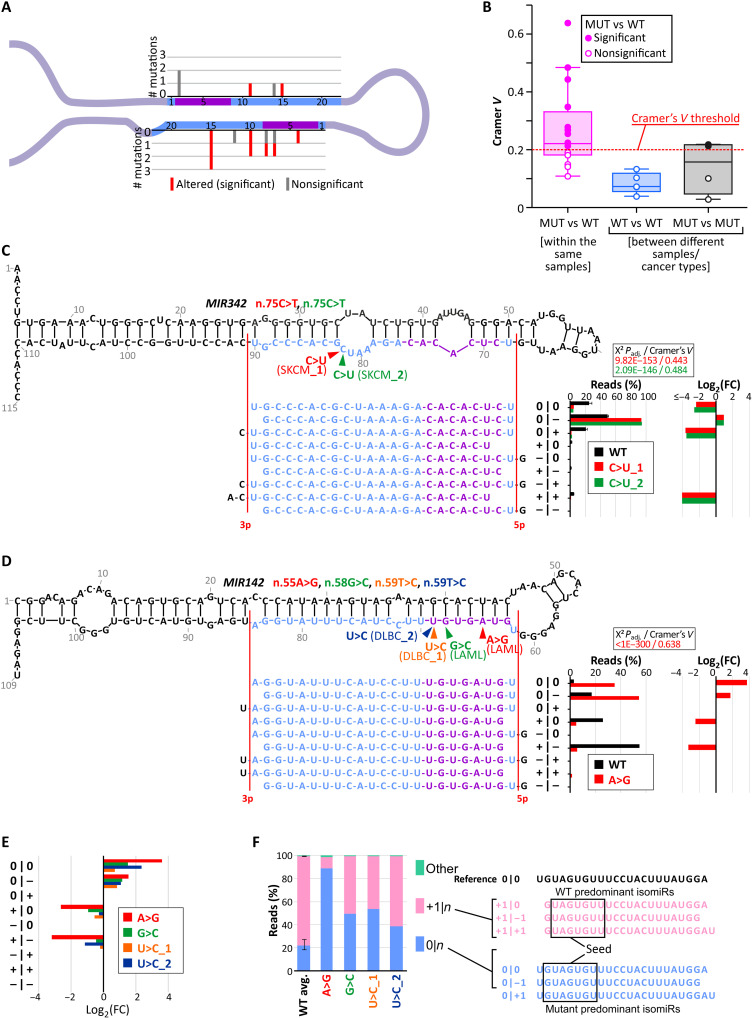
Effects of miRNA gene mutations on isomiR profiles (Experiment_2). (**A**) Distribution of the analyzed mutations. (**B**) Distribution of Cramer’s *V* values for differences in isomiR profiles between MUT and WT alleles from the same samples, WT alleles of the same miRNAs from different samples, and MUT alleles of the same type from different samples. (**C**) Comparison of WT and n.75C>T miR-342-3p isomiR profiles in two SKCM samples. Above: 2D structure of the *MIR342* precursor with analyzed mutations indicated with arrowheads; below: miR-342-3p isomiRs representing particular isomiR classes (indicated on the right); on the right: fraction of particular isomiR classes of the WT (black bars indicating an average of two samples; error bars indicating upper and lower values) and the n.75C>T MUT alleles (red and green bars from SKCM_1 and SKCM_2 samples, respectively); further right: log_2_(FC) of a particular isomiR class fraction of the MUT alleles against the WT alleles. (**D**) Comparison of WT and n.55A>G miR-142-3p isomiR profiles in the acute myeloid leukemia (LAML) sample. Above: 2D structure of the *MIR142* precursor with the indicated positions of the four mutations; others as in (C) for mutation n.55A>G. (**E**) Log_2_(FC) values of isomiR classes of four mutations [indicated in (D); against WT] located in the miR-142-3p seed. (**F**) Fractions of isomiR classes grouped only on the basis of the 5p-miRNA end (5p-isomiRs). The first bar represents the average value (from four samples) for the WT allele, and the following bars represent four *MIR142* MUT alleles. The error bar on the WT line indicates extreme values of the isomiR classes observed for the WT allele in four samples with mutations. On the right: sequences of isomiRs included in the 0|*n* and +|*n* classes.

In Experiment_2, to investigate the impact of mutations on the precision of DROSHA/DICER1 cleavage, i.e., generated isomiRs, we compared the isomiR profiles of particular miRNAs generated from wild-type and mutant alleles (*n* = 16; table S3). To compare the distribution of the corresponding wild-type and mutant miRNAs, we classified all the miRNA reads into nine isomiR classes categorized on the basis of the position of their ends (5p|3p) upstream (+) or downstream (−) against the corresponding ends annotated in miRBase (0|0) [as proposed previously (*38*) and graphically illustrated in fig. S2]. Ten of the 16 (63%) tested mutations induced significant (chi-square adjusted *P* < 1 × 10^−11^) changes in isomiR profiles and met the criterion of Cramer’s *V* > 0.2, indicating at least a moderate relationship of the mutations with the isomiR profile (table S3). Among the identified mutations, three indicate a strong effect on the isomiR profile (Cramer’s *V* > 0.4). The mutations affecting isomiR profiles are roughly equally distributed along mature miRNA sequences (Fig. 3A). To determine whether Cramer’s *V* value reliably distinguishes changes induced by mutations from random variation in isomiR distributions, we also compared isomiR profiles of the same mutants and wild-type alleles of the same miRNAs in different samples. As shown in Fig. 3B, the Cramer’s *V* values are much lower for pairs of the same mutants or wild-type alleles than for pairs of corresponding mutant and wild-type alleles from the same samples, even though the former come from different samples or even different cancer types. Thus, a Cramer’s *V* threshold of 0.2 accurately distinguishes significant changes from random variability. Most of the identified mutations induce changes of more than twofold in at least one isomiR class and, in most cases, substantially (>20%) change the level of the main isomiRs (Fig. 3, C to E, and fig. S3). Notably, six of the mutations induced changes predominantly at the 5p end, resulting in a shift in the miRNA seed sequences, which have a direct impact on target recognition.

Among the mutations with the strongest effect on the isomiR distribution was n.75C>T in *MIR342*, located at the 15th nucleotide of miR-342-3p, which shortened the miRNA at the 3p end and thus severely reduced (more than fourfold) the fraction of the canonical (0|0) and 0|+ isomiRs in favor of the 0|− isomiR (Fig. 3C). This mutation was identified in two SKCM samples. The very similar isomiR profile in two independent samples with this mutation (Cramer’s *V* > 0.2) proves the validity of the results and the specificity of the changes induced by the mutation.

An example of a mutation affecting the 5p end of the generated miRNAs is n.55A>G in *MIR142*, located at the third nucleotide of the miR-142-3p seed (Cramer’s *V* = 0.638). This mutation elongates the 5p end of the generated isomiRs by 1 nt, severely reducing the level of the +|− and +|0 isomiRs [predominant in the wild-type allele; log_2_(FC) = −3.3 and −2.5, respectively] and increasing the level of the 0|− and 0|0 isomiRs [log_2_(FC) = 1.7 and 3.7, respectively; Fig. 3D]. A similar shift (in favor of isomiRs that are longer at the 5p end) is induced by three other mutations located at nearby positions in the miR-142-3p seed [n.58G>C and n.59T>C (2×; in two samples)], although the strength of the effect varies depending on the mutation and is not formally significant (Cramer’s *V* ~ 0.18) in all cases (Fig. 3E). We subsequently combined all the isomiRs of the *MIR142* mutant samples on the basis of particular changes at the 5p end (5p-isomiRs; ignoring changes at the 3p end). As shown in Fig. 3F, the predominant isomiR class expressed from the wild-type allele (accounting for ~80% of all four samples) consists of isomiRs +1|*n* shortened by 1 nt at the 5p end. The +1|*n* isomiRs are expressed at much lower levels in the mutant alleles, ranging from 9 to 61%, depending on the mutation. In contrast, the mutant isomiRs consisted of a greater fraction (38 to 89%) of isomiRs with a canonical 5p end (0|*n*), accounting for only ~20% of the wild-type isomiRs (Fig. 3F). This shift of the 5p-miRNA end (+1|*n* to 0|*n*) results in an additional change in the seed sequence that is independent of the point changes directly introduced by the individual mutations. The remaining mutations that significantly affect isomiR distribution are shown in fig. S3.

### Analysis of the effect of miRNA gene mutations by comparing miRNAs in samples with the mutation versus samples without the specific mutation

To extend the analysis to mutations located in other parts of miRNA genes (not only in mature miRNAs that can be observed at the RNA level), we used another approach, in which we analyzed the effect of mutations by comparing miRNAs expressed from a mutated gene in a sample with the mutation with other samples of the same cancer type that lack the mutation. However, as the power of such an approach is strongly limited because of the dilution of the mutation by the normal allele and the contamination of cancer samples with normal (noncancerous) cells, as well as because of very high variation of cancer samples, we used the very restrictive significance criteria (see Materials and Methods), sacrificing sensitivity (minimizing the potential of false-positive results) to focus on identifying single mutations with the most profound effects.

**Fig. 4.**
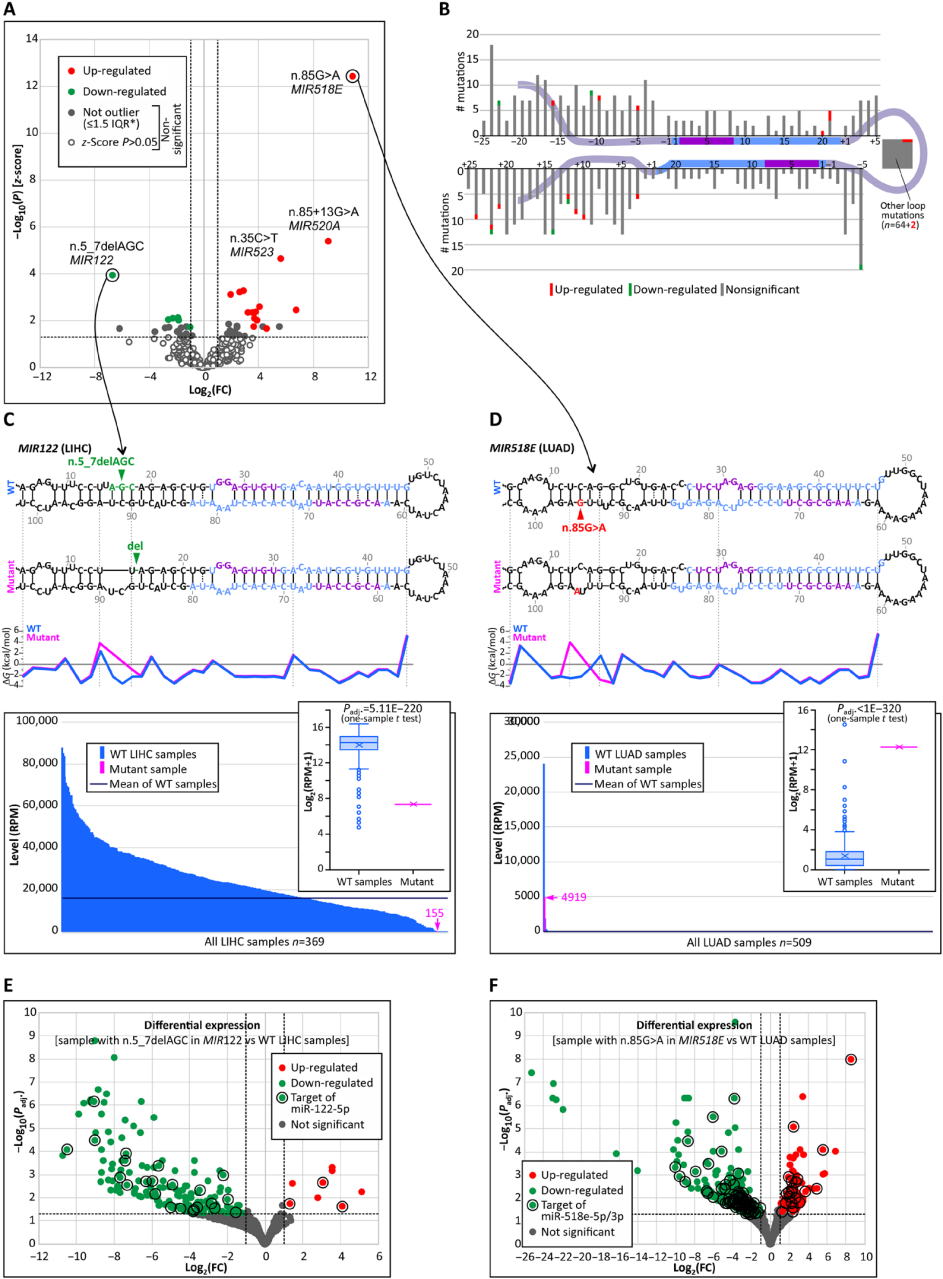
Effects of miRNA gene mutations on miRNA levels (Experiment_3). (**A**) Volcano plot showing log_2_(FC) values of the miRNA level in samples with mutations in comparison to the average miRNA level in samples (of the same cancer type) without the mutation against −log_10_(*P*) of the *z*-score. Each dot represents a mutation. Red, green, and gray (open and closed) dots represent mutations that cause increases, decreases, and no significant change, respectively. The closed gray dots represent mutations that are not outliers in the miRNA level distribution. (**B**) Distribution of the analyzed mutations. (**C**) Comparison of the *MIR122* level in the sample with the n.5-7delAGC mutation with other LIHC samples; from the top: (i) 2D structures of the *MIR122* WT and MUT precursors, (ii) the juxtaposed graph comparing the dG values of the corresponding base pairs/structural motifs of the WT and MUT precursors, and (iii) the graph showing the distribution of the miR-122 (5p + 3p) level in the LIHC samples (pink and blue bars indicate the sample with the mutation and WT samples, respectively); inset: box-and-whisker plot showing log_2_-transformed levels of the MUT and WT samples. (**D**) Comparison of *MIR518E* levels in the sample with the n.85G>A mutation versus other LUAD samples [the panel scheme as in (C)]. (**E**) Volcano plot illustrating differential expression analysis of a single sample with n.5_7delAGC in *MIR122* versus WT LIHC samples; red, green, and gray dots indicate up-regulated, down-regulated, and not significantly changed genes, respectively. The predicted direct targets of miR-122-5p are indicated on the graph in black circles. (**F**) Differential expression analysis of a sample with n.85G>A in *MIR518E* versus WT LUAD samples. The predicted direct targets of miR-518e-5p and miR-518e-3p are indicated on the graph in black circles [others as in (E)].

In Experiment_3, to identify mutations that affect the level of miRNAs, we compared levels of miRNAs in samples with mutations to the average levels of the miRNAs in samples without mutations. As shown in Fig. 4A, of the 682 mutations (648 unique mutations) that fulfilled the criteria of the analysis, 21 significantly affected the miRNA level, including 15 mutations that increased the level and 6 mutations that decreased the level (Fig. 4 and table S4). The observed excess of mutations increasing the level of miRNA (compared to Experiment_1) is likely attributable to the reduced statistical power to detect down-regulation relative to up-regulation. As shown in Fig. 4B, mutations affecting miRNA levels are located in all subregions of the miRNA precursors and are roughly equally distributed along the sequence. Among the examples of mutations affecting miRNA levels are mutation n.5-7delAGC in the 5p flank in *MIR122*, which causes a decrease in the miRNA level [log_2_(FC) < −6] (Fig. 4C), and mutation n.85G>A, which is located in the 3p flank in *MIR518E*, which leads to a marked increase in the miRNA level [log_2_(FC) > 10] (Fig. 4D). Both mutations affect the predicted two-dimensional (2D) structure and stability of the miRNA precursors by relaxing the duplex structure near the mutation site [increase in change in Gibbs free energy (ddG) [mut-wt] = 7.9 and 4.6 kcal/mol, respectively]. Moreover, both mutations alter the expression of numerous genes, including the predicted targets of the relevant miRNAs (Fig. 4, E and F, and table S5).

**Fig. 5.**
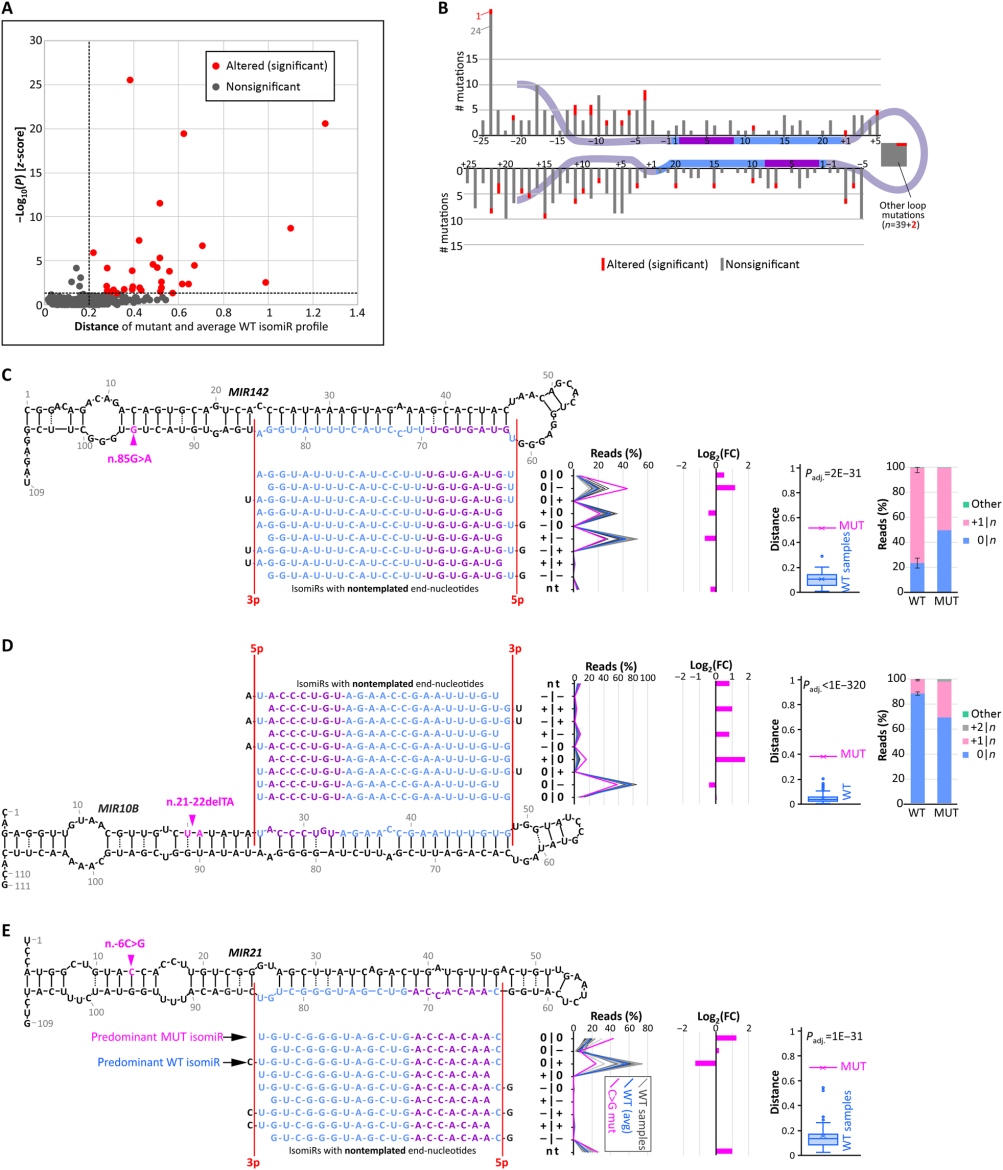
Effects of miRNA gene mutations on isomiR profiles (Experiment_4). (**A**) Scatterplot showing the distances between the isomiR profiles of the MUT samples and their corresponding WT samples against the −log_10_(*P*) of the *z*-scores of these distances. Each dot represents a mutation; red and gray dots represent mutations that significantly affect the isomiR profiles and not causing significant changes, respectively. (**B**) Distribution of the analyzed mutations along the consensus miRNA precursor structure. (**C**) Comparison of WT and n.85G>A miR-142-3p isomiR profiles in DLBC samples. From the left: 2D structure of the *MIR142* precursor with the indicated position of the mutation and juxtaposed miR-142-3p isomiRs assigned to particular isomiR classes (indicated on the right), a fraction of particular isomiR classes of the WT (average in blue and individual samples in grayscale) and the n.85G>A MUT (pink), log_2_(FC) of particular isomiR class fractions of the MUT sample against the average of WT samples, a distance of the MUT and WT isomiR profiles from the average WT profile, and fractions of isomiR classes grouped only on the basis of the 5p-miRNA end (5p-isomiRs). (**D**) Comparison of WT and n.21-22delTA miR-10b-5p isomiR profiles in COAD samples [panel scheme as in (C)]. (**E**) Comparison of WT and n.-6C>G miR-21-3p isomiR profiles in DLBC samples [panel scheme as in (C) except for the lack of a chart showing fractions of 5p-end isomiRs, as the mutation affects only the 3p end].

In Experiment_4, to investigate the effect of mutations on the precision of DROSHA/DICER1 cleavage, for each mutation, we compared the isomiR profiles of a given miRNA in the sample with the mutation and corresponding samples without mutations. Among the 420 mutations (397 unique mutations) that fulfilled the criteria, 32 mutations significantly affected the isomiR profile in at least one strand (Fig. 5A and table S6). Mutations affecting the isomiR profile are located in all subregions of the miRNA precursors and are roughly equally distributed along the sequence (Fig. 5B).

Among the mutations affecting the isomiR profile is n.85G>A, which is located in the 3p flank of *MIR142* (Fig. 5C). The mutation leads to extension by 1 nt of the 5p end of the generated miR-142-3p isomiRs, severely reducing the level of the +|0 and +|− isomiRs (predominant in the wild-type samples; log_2_(FC) = −0.5 and −0.7, respectively) and increasing the level of the 0|0 and 0|− isomiRs [log_2_(FC) = 0.5 and 1.2, respectively]. Other mutations located in different parts of *MIR142* induced a similar shift in the isomiR profile [as analyzed in Experiment_2, compare the isomiR profile graph in Fig. 3 (D to F)]. Another mutation that alters the isomiR profile is n.21-22delTA in *MIR10B*, which is located in the 5p flank of the gene (Fig. 5D). The mutation causes the extension of the 5p end of miR-10b-5p by 1 or 2 nt, leading to a reduction in the fraction of 0|*n* isomiRs [log_2_(FC) = −0.35] in favor of +1|*n* and +2|*n* isomiRs [log_2_(FC) = 2.5 and >>5, respectively]. Another example is n.-6C>G in *MIR21*, which is located in the 5p flank of the gene (Fig. 5E). The mutation induces shortening by 1 nt of the 3p miRNA at its 3p end, severely reducing the fraction of 0|+ isomiRs [log_2_(FC) = −1.3] predominant in wild-type samples in favor of the canonical (0|0) isomiR [log_2_(FC) = 1.2]. As shown in the volcano plots (fig. S4), some genes are differentially expressed in the mutated samples. However, the relationship between the differentially expressed genes and the observed isomiR shifts cannot be directly determined. The isomiR profiles of other mutations that significantly affect isomiR profiles are presented in fig. S5.

**Fig. 6.**
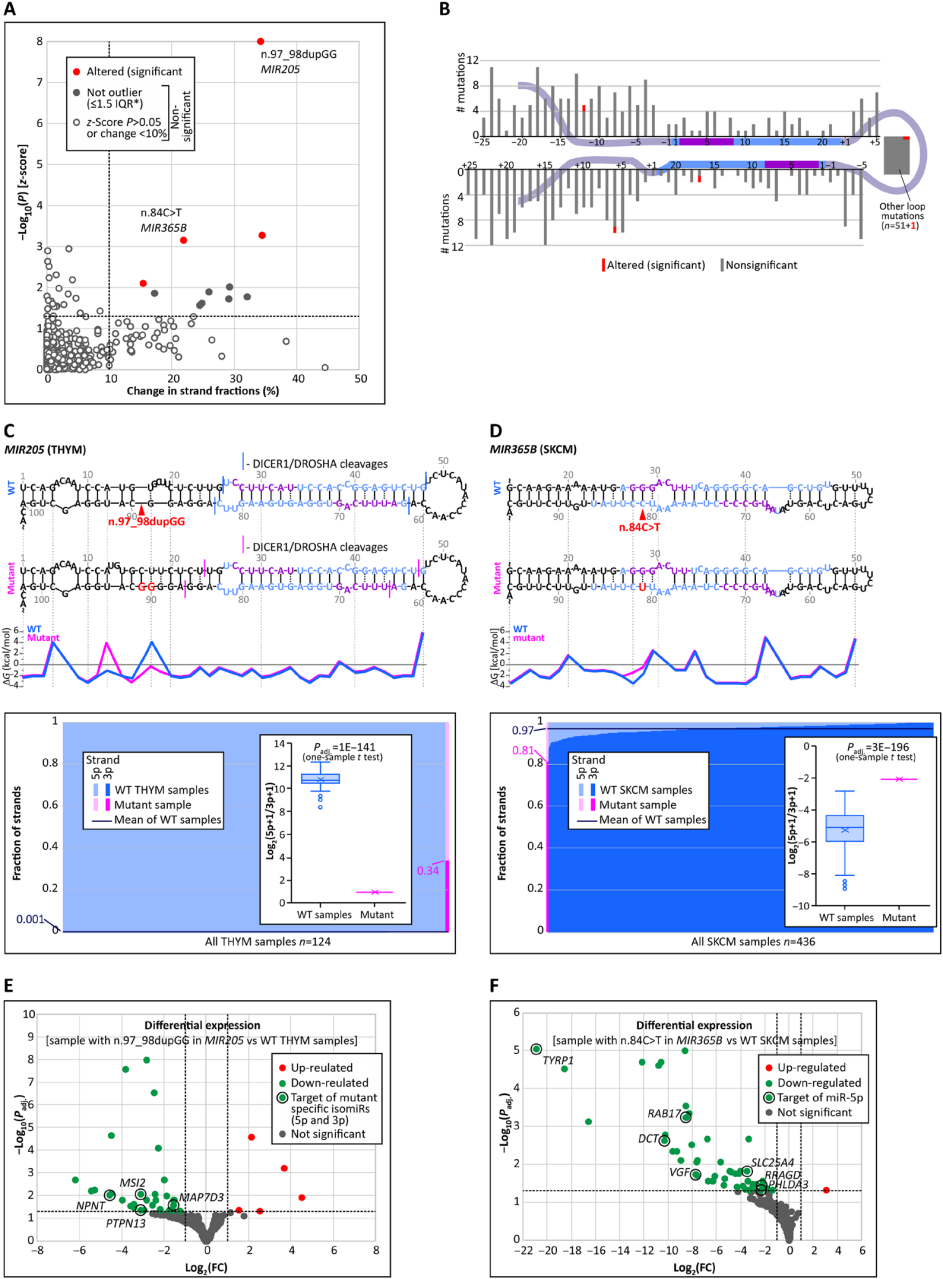
Effects of miRNA gene mutations on the balance of miRNA strands (Experiment_5). (**A**) Relationship between the change in the 5p/3p strand fraction between MUT and WT samples and the −log_10_(*P*) of the *z*-score for strand balance [log_2_(5p + 1/3p + 1)] in a sample with a mutation compared with samples of the same cancer type without mutation. Each dot represents a mutation. Red and gray (open and closed) dots indicate mutations affecting 5p/3p strand balance and not inducing significant changes, respectively. The closed gray dots represent mutations that are not outliers in the strand balance distribution. (**B**) Distribution of the analyzed mutations. (**C**) Comparison of 5p/3p strand balance in the sample with the n.97-98dupGG mutation in *MIR205* versus other THYM samples without mutation; from the top: (i) 2D structures of the *MIR205* WT and MUT precursors, (ii) the juxtaposed graph comparing dG values of corresponding WT and MUT base pairs/structural motifs, and (iii) the graph showing the proportion of miR-205-5p and miR-205-3p strands in the MUT sample (pink) and in WT samples of THYM (blue); inset: box-and-whisker plot showing log_2_-transformed 5p/3p strand balance in the MUT sample versus distribution of the balance in other THYM samples. (**D**) Comparison of 5p/3p strand balance in the sample with the n.84C>T mutation in *MIR365B* versus other SKCM samples [the panel scheme as in (C)]. (**E**) Volcano plots illustrating differential expression analysis of a single sample with n.97_98dupGG in *MIR205* versus WT THYM samples. The direct targets of hsa-mir-205-5p −2|*n* and hsa-mir-205-3p +2|*n* isomiRs are indicated in black circles. (**F**) Differential expression analysis of a sample with n.84C>T in *MIR365B* versus WT SKCM samples [scheme of the panel as in (E)]. The direct targets of the hsa-mir-365b-5p 0|*n* isomiR are indicated by black circles.

In Experiment_5, we analyzed the effect of mutations on miRNA strand balance, i.e., the ratio of miRNAs derived from 5p and 3p miRNA arms of the precursor, compared between mutant and wild-type samples as log_2_(5p + 1/3p + 1). As shown in Fig. 6A, from the 515 mutations (490 unique mutations) fulfilling the criteria (for details, see Materials and Methods), 4 mutations significantly affected the miRNA strand balance, including 1 mutation located in each of the following regions: 5p flank, 3p flank, loop, and miRNA duplex (Fig. 6, A and B, and table S7).

The most notable example is the duplication of two Gs (n.97_98dupGG) in the 3p flank in *MIR205* identified in thymoma (THYM). In all the THYM samples, miR-205-5p was the predominant strand, accounting for more than 99.7% of all the reads (similar to other TCGA cancers, as well as other tissues reported in different databases). In contrast, in the mutant sample, the fraction of miR-205-5p decreased substantially (down to 65%), resulting in a marked increase (>100×, up to 35%) of the miR-205-3p fraction (Fig. 6C). It is noteworthy that the observed change of the 5p/3p balance is detectable, even though the fraction of the mutant allele (at the DNA level) accounts for only 19%, which suggests that the actual effect of the mutant allele is much stronger (this comment also applies to other mutations mentioned in this section). A closer look at the miRNAs generated from the 5p and 3p arms revealed that, in addition to changing the 5p/3p balance, the mutation also shifted (by 1 to 3 nt toward the base of the precursor hairpin) the predominant DROSHA/DICER1 cleavage sites, generating abnormal miRNAs (Fig. 6C). To determine whether this marked strand/isoform imbalance (the mutant-specific miRNA) affects gene expression, we performed differential expression analysis and identified 31 and 5 genes whose expression was down-regulated and up-regulated, respectively, in the sample with the *MIR205* mutation. Among the down-regulated genes, four targets (predicted by TargetScan and/or mirDB) of mutant-specific miR-205-5p (−2|−1) and 3p (+2|+3) were identified (table S5 and Fig. 6E).

An example of a mutation that disturbs the balance in the opposite direction (in favor of the 5p strand) is n.84C>T in *MIR365B*, which is located in the 3p strand of the miRNA duplex (Fig. 6D). This mutation increases the level of the 5p strand more than fivefold, from 3 to 19%. As the mutation destabilizes the miRNA duplex at the 5p end of miR-365b-5p (replaces G:C with the G:U pair and increases the duplex free energy by 2 kcal/mol), its effect of favoring the 5p strand over the 3p strand is consistent with the principle of greater preference/stability (more efficient loading into the miRNA-induced silencing complex) of the strand with a more relaxed (less stable) 5p end (Fig. 6D) (*39*). As shown in Fig. 6F, many genes were down-regulated in the mutated sample, and seven of these genes were predicted targets of miR-365b-5p, the fraction of which was increased in the mutant sample. The other two mutations that affect the strand balance are shown in fig. S6.

Together, of 703 mutations selected for at least one experiment (fig. S1), we identified 87 mutations that significantly altered at least one aspect of miRNA processing (functional mutations; 9 mutations affected more than one function). Notably, functional mutations were overrepresented in the group of mutations classified as homozygous [20 of 88 (22.7%) versus 67 of 615 (10.9%, for nonhomozygous mutations); fold enrichment = 2.1; Fisher’s exact test, *P* = 0.003]. This enrichment may reflect a higher statistical power to detect the effect of homozygous mutations, which have an increased fraction of mutated alleles, and/or suggest the cancer relevance of these mutations. However, the latter would require functional validation in a model relevant to the specific cancer type and a mutated miRNA gene. In addition, given that the measurement of mutation allelic fraction (homozygosity) and the identification of functional mutations were completely independent, the strong association between them further confirms the reliability of the identified functional mutations.

### Impact of miRNA gene mutations on the structure of miRNA precursors

**Fig. 7.**
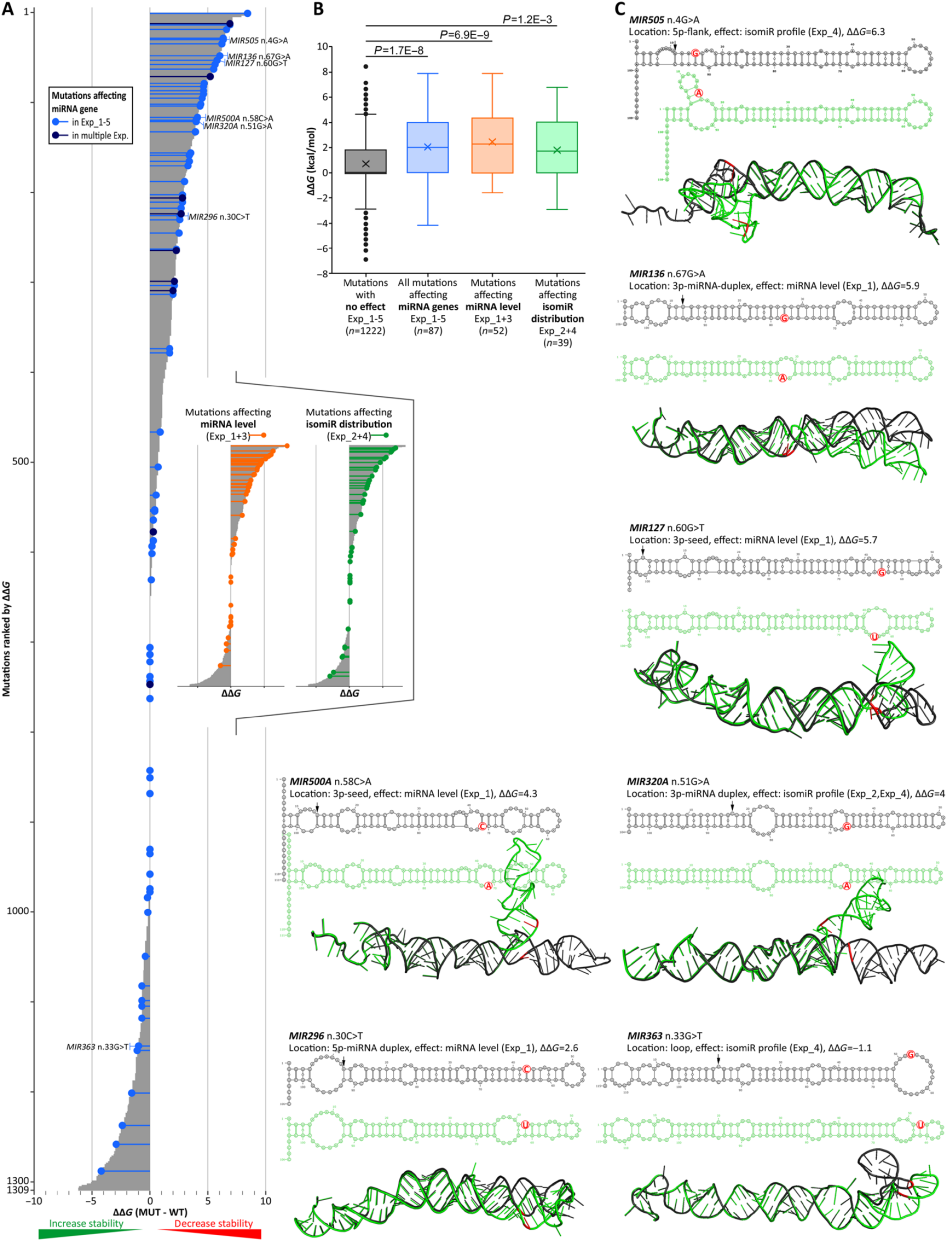
Impact of miRNA gene mutations on the stability and structure of miRNA precursors. (**A**) Chart showing the effect of mutations on the stability of miRNA precursor structures, i.e., the increase or decrease in dG between mutated and corresponding WT miRNA precursors, expressed as ddG. The graph shows all mutations preselected for the analysis (*n* = 1309; see Fig. 1) sorted from the highest (destabilizing mutations) to lowest (stabilizing mutations) ddG values. Light and dark blue lollipops represent mutations significantly affecting the functioning of miRNA genes in one or more experiments, respectively. In the inset, flow charts separately highlight only mutations affecting the level of miRNA (orange lollipops, Experiment_1 and Experiment_3) and isomiR profiles (green lollipops, Experiment_2 and Experiment_4), respectively. (**B**) Box plots comparing the effect of mutations on miRNA precursor stability, i.e., the distribution of ddG values for mutations for which no effect on miRNA gene function was detected (gray box), mutations for which an effect was detected in at least one experiment (blue box), mutations affecting miRNA levels (orange box), and mutations affecting the isomiR profiles (green box). (**C**) Effect of representative mutations [indicated in (A)] affecting the functioning of miRNA genes on the secondary (above) and spatial (below) structures of miRNA precursors. WT and MUT structures are shown in dark gray and green, respectively; mutation positions are indicated in red.

Although mutations can affect the functioning of miRNA genes through various mechanisms, the most direct/common impact seems to be modifying the structure of miRNA precursors. Therefore, to preliminarily estimate the effects of mutations on the structure of miRNA precursors, we modeled and compared the wild-type and mutant precursor structures of all the tested miRNA genes (*n* = 1309). Most mutations increase the change in Gibbs free energy (dG) value between mutant and wild-type precursors, i.e., decrease the stability of the precursor structures (table S8). The average dG value of the mutant structures was 0.77 kcal/mol greater than that of the corresponding wild-type structures (*P* = 1.5 × 10^−35^; paired *t* test), and there was an excess of mutations increasing the dG value (destabilizing the precursor structure) over mutations decreasing the dG value (Fig. 7A). Further comparisons revealed that the ddG was significantly greater for functional mutations identified in Experiment_1 to Experiment_5 than for the remaining mutations (*t* test, *P* = 1.73 × 10^−08^). As shown in Fig. 7B, the functional mutations cluster at the top of the ddG ranking. The ddG for functional mutations is independent of the functional effect of the mutation; it applies to both mutations affecting miRNA levels (*t* test, *P* = 6.92 × 10^−09^) and mutations affecting isomiR distributions (*t* test, *P* = 0.0012) (Fig. 7B; because of the low number of mutations, we did not test mutations affecting 5p/3p strand balance). The relationship between the results of structural and functional analyses further confirms the reliability of the identified functional mutations (results of Experiment_1 to Experiment_5), as both analyses are completely independent. Examples of mutations affecting the miRNA precursor structures are shown in Fig. 7C.

### Experimental analysis of mutations in *MIR142* and *MIR205*

**Fig. 8.**
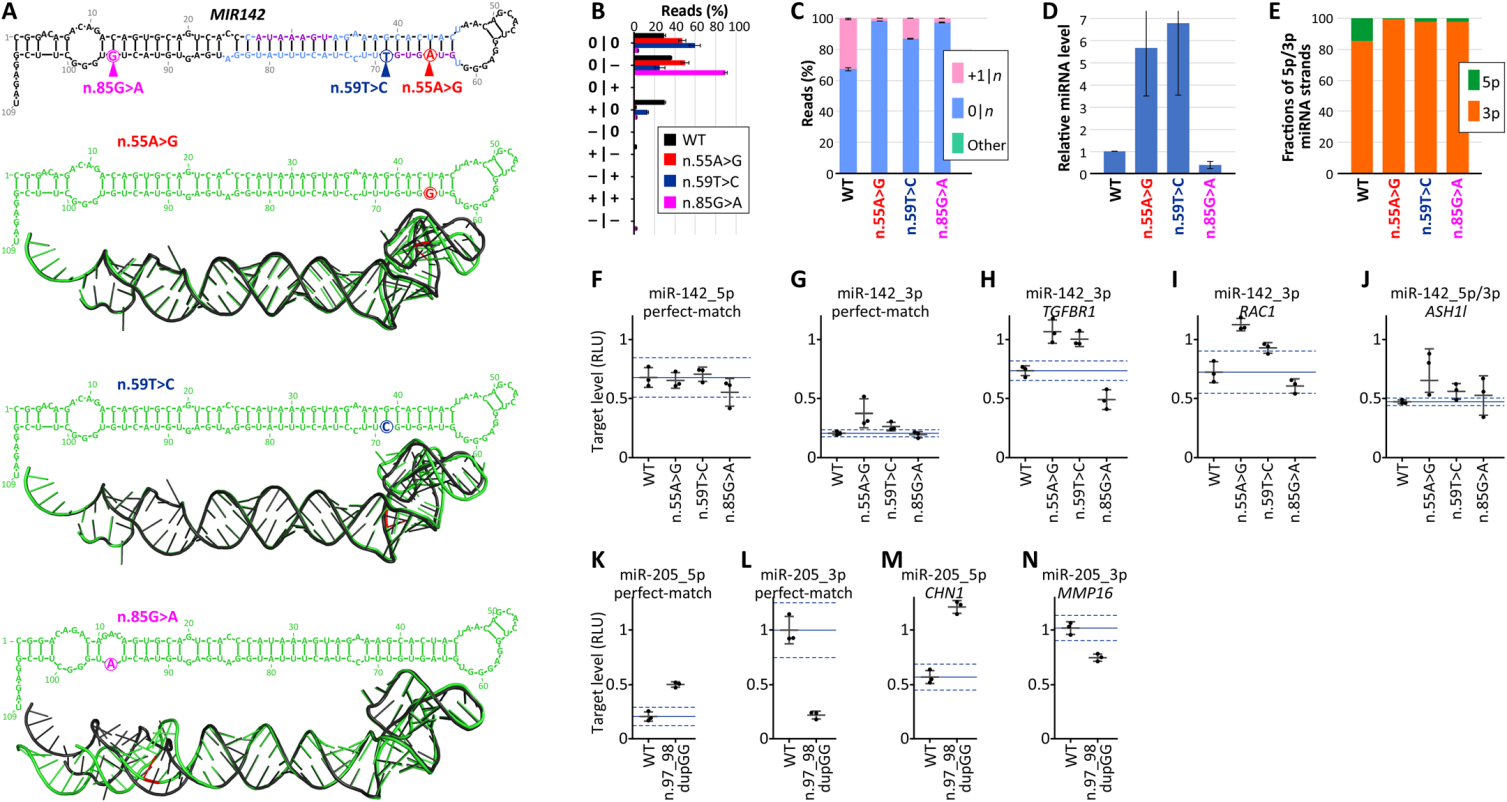
Experimental analysis of mutations in *MIR142* and *MIR205*. (**A**) Localization and structural effects of mutations in *MIR142*, n.55A>G, n.59T>C, and n.85G>A (mutation color scheme as in Figs. 3D and 5C). (**B**) Fraction of specific miR-142-3p isomiR classes in WT (black bars) compared to n.55A>G (red), n.59T>C (dark blue), and n.85G>A (magenta) MUT samples expressed in HEK293T cell lines transfected with corresponding expression vectors. Bars represent the averages, with error bars showing standard deviation. (**C**) Fractions of 0|*n*, +1|*n*, and other miR-142-3p isomiR classes grouped on the basis of the 5p-miRNA end. (**D**) Level of miR-142 in HEK293T cells after transfection with WT and MUT expression vectors. The miRNA level, including 5p and 3p strands, was normalized against the internal control miRNA (misafe) and the average WT miR-142 levels. (**E**) Proportion of WT and MUT miR-142 reads derived from 5p and 3p miRNA strands. (**F** to **J**) Results of dual-luciferase assay demonstrating the effectiveness of *MIR142* WT and MUT samples in silencing miR-142-5p perfect-match target (F), miR-142-3p perfect-match target (G), TGFBR1 (3p) target (H), RAC1 (3p) target (I), and ASH1L (5p/3p) target (J). Target levels are expressed in relative luciferase units (RLU). Each target’s levels are shown as individual black dots, with vertical lines indicating averages and error bars showing standard deviations. The blue vertical line indicates the average target level in the WT cell line, while dashed blue lines indicate a WT *z*-score equal to 2 above and below the average WT. The experiments were performed in triplicate. (**K** to **N**) Results of dual-luciferase assay demonstrating the effectiveness of *MIR205* WT and n.97_98dupGG MUT in silencing miR-205-5p perfect-match target (K), miR-205-3p perfect-match target (L), CHN1 (5p) target (M), and MMP16 (3p) target (N). Graph schemes are shown in (F) to (J). The location and structural effect of n.97_98dupGG are shown in Fig. 6C.

To experimentally test the effect of mutations in miRNA genes, we selected three mutations in *MIR142* (n.55A>G, n.59T>C, and n.85G>A) and one mutation in *MIR205* (n.97_98dupGG), which, in computational analyses of TCGA data, showed a significant effect on the function of miRNA genes. *MIR142* mutations are located in different parts of the gene and cause distinct effects on the structure of the miR-142 precursor (Fig. 8A). *MIR142* is known to be recurrently mutated in various blood cancers, especially B cell lymphomas, and mutations in *MIR142* are suggested to be potential cancer drivers [reviewed in (*9*)].

First, we performed sRNA-seq on RNA extracted from human embryonic kidney (HEK) 293T cells transfected with plasmids expressing either the wild-type or mutant versions of pre-miR-142. The analysis revealed significant differences in the isomiR profiles between wild-type and mutant samples (Fig. 8B). Although there were some differences in the proportions of specific isomiRs between TCGA cancer samples and HEK293T cell lines—likely due to biological differences between the models—the overexpression experiment consistently showed a decrease in +1|*n* isomiRs and an increase in 0|*n* isomiRs across all mutant-expressing versus wild-type cell lines (Fig. 8C; compare to Figs. 3F and 5C). Further analysis of the sRNA-seq data showed a substantial increase in the relative level of miR-142 (combining miR-142-5p and miR-142-3p) in the n.55A>G and n.59T>C cell lines and a decrease in miR-142 levels in the n.85G>A cell line (Fig. 8D). In addition, all three mutations shifted the strand balance toward miR-142-3p, substantially decreasing the level of miR-142-5p (Fig. 8E).

Subsequently, we used a dual-luciferase reporter assay to evaluate the impact of mutations on the silencing efficiency of miRNA targets (target sequences and miRNA-target pairing are presented in figs. S7 and S8). As shown in Fig. 8 (F to J), mutants n.55A>G and n.59T>C reduce the silencing efficiency of the perfect-match target of miR-142-3p (Fig. 8F); two natural targets of miR-142-3p, *RAC1* and *TGFBR1* (Fig. 8, H and I); and *ASHL1* (Fig. 8J), which is the predicted target for both miR-142-3p and miR-142-5p. Conversely, n.85G>A enhances the silencing of the *TGFBR1* target and does not affect the silencing efficiency of other targets. None of the mutants decrease the silencing of the perfect-match target of miR-142-5p, which was already relatively low. In summary, the experimental analysis of mutations in *MIR142* confirmed the effect of the mutants on the miR-142-3p isomiR profile, showed their effect on the overall level of miR-142 and the miRNA 5p/3p strand balance, and demonstrated the reducing effect of two mutations on the silencing of miR-142-3p targets.

Next, we performed a dual-luciferase test of the mutation n.97_98dupGG located in the 3′ flanking sequence of *MIR205*, identified in Experiment_5 as a variant that severely affects the 5p/3p strand balance in favor of the miR-205-3p strand (Fig. 6C). Under natural conditions and in wild-type cancer samples, the predominant (mature) strand of *MIR205* is miR-205-5p, while miR-205-3p is almost absent. As shown in Fig. 8 (K to N), the mutation decreases the silencing of both perfect-match and natural (*CHN1*) targets of miR-205-5p and increases the silencing of perfect-match and natural (*MMP16*) targets of miR-205-3p, which is consistent with the effect observed in Experiment_5.

## DISCUSSION

Thanks to the genetic code, predicting the consequences of mutations and distinguishing deleterious from neutral mutations in coding sequences are relatively straightforward (although not trivial). On the other hand, owing to modern sequencing approaches, such as whole-genome sequencing, thousands of previously unidentified genetic variants, primarily located in noncoding sequences, are being identified in each sequenced sample. Unfortunately, because of the heterogeneity of noncoding elements and a lack of appropriate “codes” (rules), predicting the consequences of mutations or even estimating the likelihood of deleteriousness for mutations in the best-defined noncoding elements, such as miRNA genes, is nearly impossible. Consequently, the vast majority of sequence variants identified in the noncoding genome is assumed to be neutral or variants of unknown significance and is ignored. To overcome these limitations and ultimately develop “codes” to predict the consequences of variants in noncoding elements, more data need to be collected on the impacts of mutations on the functionality of these elements.

Among the most well-recognized and highly conserved noncoding elements are miRNA genes, particularly their ~100-nt-long regions encoding hairpin-structured miRNA precursors (pre-miRNAs). Although analysis of genetic variation in the noncoding genome has long been neglected, numerous variants in miRNA genes have been extracted from whole-genome datasets. Moreover, a few miRNA gene mutations have been implicated in Mendelian diseases, including mutations in *MIR96* in nonsyndromic hearing loss (*11*), mutations in *MIR184* in different hereditary eye diseases (*12*), mutations in *MIR204* in retinal dystrophy (*13*), and mutations in *MIR140* in skeletal dysplasia (*14*). There are also a few miRNA genes whose mutations have been suggested to be potential cancer drivers, including the *MIR15A/MIR16-1* cluster in chronic lymphocytic leukemia (CLL), *MIR142* in different blood cancers, *MIR122* in liver carcinoma, and *MIR21* in various cancers (*7*, *15*, *40*–*43*). Nevertheless, a larger-scale analysis of the molecular consequences of miRNA gene variants has never been performed, and almost all previous studies have focused on mutations identified in the seed sequences (*11*, *13*, *14*, *17*, *18*, *44*–*47*). Only a few studies have investigated the impact of variants in other regions of miRNAs [summarized in (*9*)] (*19*, *20*, *25*, *48*–*52*). Moreover, almost all of these studies have investigated only the impact on the miRNA level. To date, the only and largest systematic analysis of sequence variants in miRNA genes, performed more than 15 years ago, has examined the effects of 24 common SNPs located in different parts of miRNA precursors. Using several cellular functional assays, the study revealed that most of the variants disturb the proper function of miRNA genes, including the efficiency of miRNA generation and precision of miRNA processing (*20*).

Therefore, taking advantage of the large collection of mutations and corresponding transcriptome (sRNA-seq) datasets generated by the TCGA project, we performed the most comprehensive analysis to date of the impact of mutations on miRNA gene functioning, exceeding the number of variants studied in previous projects by orders of magnitude. In contrast to most previous studies, the approach allowed us to analyze the effects of mutations in their real genetic context, under natural conditions in real cells/tissues, without artificially generated models using engineered genes, overexpression, or generic cell lines. Furthermore, in contrast to most previous studies, our analysis included mutations located in all parts of the miRNA gene, not only in the seed region. An additional advantage of our study is that the vast majority of somatic mutations is randomly occurring variants. This ensures that the analysis is not biased toward more likely neutral variants, such as common SNPs, or deleterious variants, such as mutations identified in Mendelian diseases. Moreover, in addition to the analysis of the impact of mutations on the miRNA level, which has been mostly analyzed in other studies, we also analyzed the effects of mutations on the miRNA strand balance and isomiR profiles.

Together, we analyzed the effects of 703 mutations selected in multiple steps from ~7000 mutations identified in miRNA genes in TCGA samples (Fig. 1 and fig. S1). The mutations were selected on the basis of very strict criteria, ensuring that their effects may be reliably evaluated (e.g., by excluding mutations in redundant miRNA genes such as *MIR1-1* and *MIR1-2*). Nevertheless, this is the largest and most complex analysis of miRNA gene mutations performed to date. It is worth noting, however, that excluding redundant miRNA genes from the analysis does not diminish their importance. Many of these genes belong to the well-validated and highly expressed miRNAs with well-defined functions. It might be expected that mutations in redundant genes may affect their function in a manner similar to mutations in other genes, although the effect of mutations in such genes may be masked by their nonmutated copies. An example of such a mutation may be a rare germline variant n.43G>A, identified in patients with breast and gastric cancers, located in the apical loop of *MIR30C-1* in the GUG regulatory motif (*53*, *54*). It was demonstrated that the variant, through alteration of the precursor structure, enhances DROSHA processing, resulting in increased levels of miR-30c (*55*). Also, the exclusion of longer indels and double mutations was primarily due to the potential uncertainty they could introduce into the analysis. However, this does not rule out their potential impact on miRNA genes. Most longer indels are likely to significantly disrupt the structure of miRNA precursors and, as a result, the functionality of miRNA genes, whereas double mutations may represent two allelic mutations, indicating a cancer-relevant role of the mutated miRNA gene.

First, by comparing the levels of mutant and wild-type alleles at the genomic (DNA) and transcriptomic (miRNA) levels, we showed that most miRNA gene mutations (at least those located in the miRNA duplex) affect the miRNA level (32 of 53) and/or precision of DROSHA/DICER1 cleavages (isomiR profile, 10 of 16), i.e., they are deleterious for the proper functioning of miRNA genes at the molecular level. Additional mutations, which are located in the whole pre-miRNA and affect miRNA gene functionalities, including miRNA levels (*n* = 21), isomiR profiles (*n* = 32), and miRNA strand balance (*n* = 4), were detected by comparing the level and sequence of miRNAs in the mutated versus nonmutated samples. However, because of the dilution of mutants with the wild-type allele, contamination of cancer samples with normal tissues, cancer heterogeneity, and high variation between cancer samples, the sensitivity of the latter approach was low. Therefore, the mutations identified here likely reflect only a fraction of the functionally relevant events and should be interpreted as representative mutations rather than an exhaustive set. We can assume that, among the mutations not identified in this study as significantly affecting miRNA genes, some may still be harmful mutations for these genes or may have a functional role in cancer. However, first, to identify interesting and/or well-supported variants that are worth further investigation through more extensive, variant-specific functional and/or disease-related studies, more samples of specific cancers or other diseases need to be analyzed with a focus on noncoding variants. To further assess the effects of such mutations, allele-specific functional methods, such as luciferase reporter assays or sRNA-seq, can be used using naturally occurring or genome-edited in vitro and/or in vivo models. Furthermore, single-cell sequencing of cancer samples may provide insights into the effects of the mutation in particular subsets of cancer cells. In addition, techniques like AGO-CLIP (Argonaute cross-linking and immunoprecipitation), iCLIP (individual-nucleotide resolution cross-linking and immunoprecipitation), or single-cell RNA-seq can be used to determine the altered target set of a specific mutated miRNA. In addition, some mutations may exert long-range effects; mutations in miRNA genes can affect the structure or generation of primary miRNA precursors, while more distant mutations may affect the processing of miRNA. To analyze such effects, long-read sequencing may be used.

As shown in subsequent experiments, mutations affecting miRNA genes were dispersed roughly equally across all subregions of the genes and did not cluster or show enrichment in any specific region. These functional mutations occurred not only in the nonseed miRNA duplex (*n* = 27) and seed sequences (*n* = 14) but also in the apical loop (*n* = 10) and the flanking regions (*n* = 17 and *n* = 19 in 5′ and 3′ flanks, respectively), which contain various sequence and/or structural regulatory motifs. Among the most well-defined regulatory motifs facilitating miRNA biogenesis are the UG basal motif in the 5′ flanking region, the CNNC motif in the 3′ flanking region, the mismatched GHG motif in the lower stem of primary miRNAs, and the UGU motif in the pre-miRNA terminal loop (*29*, *56*). It was also demonstrated that sequences of ultraconserved terminal loops may play a role in miRNA biogenesis through interactions with trans-acting factors such as Lin28, KSRP, or hnRNP A1 (*57*, *58*). Examples of functional mutations identified in our study that affect regulatory motifs include (i) n.38C>G in *MIR409*, which results in the formation of a UGUG motif in the loop; (ii) n.92A>C in *MIRLET7C*, which causes the creation of a CNNC motif adjacent to the existing one in the 3′ flanking region; and (iii) n.-4C>T in *MIR134*, which produces a UG motif in the 5′ flanking region at the basal junction (fig. S9). In addition, interesting observations may include the mutation n.84C>T in *MIR365B*, which changes the stability of one side of miRNA duplexes and thus markedly reverses the miRNA strand balance. This observation is in agreement with the notion that the miRNA strand with the less stable 5p end is more favorably loaded into the RNA-induced silencing complex, becoming a more stable mature miRNA (*39*). Last, we showed that most miRNA gene mutations decrease the stability of miRNA precursors. We also showed that mutations detected as having a functional effect cause, on average, a greater decrease in miRNA precursor stability. This association suggests that at least some mutation effects are expressed via changes in the miRNA precursor structure, confirming the importance of the structure and its stability for the proper functioning of miRNA precursors.

Although most tested mutations, including those affecting the molecular functionality of miRNA genes, are randomly occurring neutral variants, single ones may still play a role in driving cancer development. This applies, in particular, to mutations in cancer-related miRNA genes, such as those annotated in the Cancer miRNA Census (*40*), including 28 miRNA genes annotated in the Cancer miRNA Census, in which mutations affecting miRNA gene functioning were detected. One possible example of such a gene is *MIR142*, which is recurrently mutated in various hematologic malignancies, including acute myeloid leukemia (LAML), CLL, DLBC, follicular lymphoma (FL), and other types of B cell lymphomas. *MIR142* was found to be the most frequently mutated miRNA gene in any cancer and overmutated in the TCGA cohort in different cancers, especially in DLBC and LAML (*7*, *59*). *MIR142* is highly expressed in lymphoid blood cells, and it has been shown that miR-142 (particularly miR-142-3p) plays an important role in hematopoiesis, regulating the development and function of different hematologic lineages as well as in hematologic malignancies (*60*). Functional studies have focused mainly on a few mutations in the miR-142-3p seed. However, these studies, conducted in various cellular and animal models, demonstrated that the mutations, through ineffective regulation of miR-142-3p targets, including *ASH1L*, increase the level of homeobox A9/A10 (HOXA9/A10), resulting in aberrant hematopoietic differentiation (*17*). This promotes myeloid lineages and suppresses lymphoid lineages, ultimately leading to leukemic transformation and LAML. Mutations synergize with mutations in *IDH2* (*44*). A gain-of-function effect for one of the mutations was also suggested (*61*). The functional effects of the seed mutations were observed despite their small impact on the level of miR-142-3p, suggesting that the mutations act predominantly by affecting the seed sequence and, thus, target recognition. This finding is consistent with the lack of effects of n.59T>C (two samples) and n.58G>C on the miR-142-3p level observed in our study (Experiment_1; Fig. 2A). A recent study published during the preparation of this manuscript also demonstrated the molecular effects of mutations located outside the 3p seed in different parts of *MIR142* overexpressed in HEK293 cells (*19*). To date, however, *MIR142* mutations have been studied only in artificially generated cellular or mouse models. Here, we demonstrated the molecular effects of *MIR142* mutations in relevant cancer samples, predominantly LAML and DLBC. We found that two mutations, n.55A>G and n.16C>A, severely affected the levels of miR-142-3p and miR-142-5p (Experiment_1; table S2). In addition, three *MIR142* mutations significantly affected the distribution of miR-142-3p isomiRs (n.55A>G and n.59T>C in Experiment_2 and n.85G>A in Experiment_4). Unexpectedly, despite being located in different parts of the precursor, all these mutations induced similar effects on the isomiR profile, leading to a decrease in +1|*n* isomiRs (predominant in wild type) in favor of 0|*n* isomiRs. A similar (although less profound) shift in the isomiR profile was also triggered by two other mutations in *MIR142* (n.58G>C and n.59T>C). Thus, all of these mutations affect the miR-142-3p seed by shifting the miRNA; in addition, three of the mutations located in the 3p seed also directly alter its sequence. Our experimental analyses have shown a similar effect of all three analyzed mutations (n.55A>G, n.59c>T, and n.85G>A) in *MIR142* to that observed in computational analysis, which results in a shift in isomiR profile.

Another well-known cancer-related miRNA gene is *MIR205*, which is important for cancer development. It has been assigned both an oncogenic role and tumor suppressor role, depending on the tissue/cancer type (*62*, *63*). Analysis of TCGA datasets revealed that *MIR205* is overmutated in different solid cancers (*7*), particularly in melanoma, suggesting the potential of these mutations as drivers; however, their impact on miRNA functioning has never been studied. Moreover, a reduced level of miR-205-5p is associated with shorter survival in patients with melanoma (*64*). In this study, we found that n.35C>T, located in the 5p arm of *MIR205* (Experiment_1), decreases the level of miR-205 in SKCM (melanoma). Two other mutations, n.30C>T and n.94G>C, identified in BLCA (bladder urothelial carcinoma) and localized in 5p and 3p flanks, respectively, affect the isomiR profiles of miR-205-5p (Experiment_4; fig. S5). Last, n.97-98dupGG, located in the 3p flank (Experiment_5; Fig. 6C), affects the structure of miRNA precursors, resulting in a shift in DROSHA and DICER1 cleavage sites and reversing the 5p/3p strand balance, reducing the fraction of miR-205-5p (dominant mature strand) in favor of miR-205-3p (passenger strand). The effect of mutation on strand balance was consistent with experimental results of silencing targets of 5p and 3p miRNAs.

Two more examples of mutations located in cancer-related genes are n.21-22delTA in *MIR10B* (*65*) and n.-6C>G in *MIR21* (*66*). Both mutations led to significant changes in isomiR profiles (Experiment_4; Fig. 5). The mutation in *MIR10B* increases the +1|*n* and +2|*n* fractions of miR-10b-5p, changing its canonical seed. In contrast, the mutation in *MIR21* affects miR-21-3p, decreasing the fraction of 0|+ in favor of 0|0 and nontemplated isomiRs.

Notably, however, most mutations identified in this study as affecting miRNA genes should not be considered physiologically functional or necessarily playing a role in cancer. By definition, only a small fraction of mutations occurring in the cancer genome (including mutations in miRNA genes) is expected to play a role in cancer and/or act as cancer-driving mutations. To prove such an effect, a specific set of functional tests focused on and adjusted to the expected impact of a particular mutation (or miRNA) under a specific cancer condition would have to be performed, which is beyond the scope of this study.

In summary, to the best of our knowledge, we performed the broadest systematic analysis of the effects of mutations in miRNA genes, in which we identified 87 mutations that significantly affect different miRNA gene functionalities, including miRNA levels, isomiR profiles, and strand balance. The strong associations between the identified functional mutations and independent measures such as ddG and mutation allelic fraction further validate the reliability of our findings. We studied the effects of mutations in natural genomic contexts, not in artificially generated models. Our results improve the understanding of the impact of genetic variants on miRNA biogenesis and may help develop tools for predicting the significance of genetic variants in miRNA genes. In general, our results (the first approach) indicate that most miRNA gene mutations, not only those located in seeds, affect the proper functioning of miRNA genes and should therefore be considered likely deleterious variants in genetic analyses.

## MATERIALS AND METHODS

### Experimental design

The aim of our study was to evaluate the effects of miRNA gene mutations identified in TCGA cancer samples on miRNAs functioning. The IDs, sequences, and genomic coordinates of miRNAs (5p and 3p strands) and miRNA precursors were obtained from miRBase version 22.1 (*5*) and used as references for mutation annotation and isomiR classification. The miRNA gene IDs were used according to HUGO Gene Nomenclature. The following cancer type names and abbreviations were used according to TCGA nomenclature: adrenocortical carcinoma (ACC), BLCA, breast invasive carcinoma (BRCA), CESC, cholangiocarcinoma (CHOL), colon adenocarcinoma (COAD), DLBC, esophageal carcinoma (ESCA), HNSC, kidney chromophobe (KICH), kidney renal clear cell carcinoma (KIRC), kidney renal papillary cell carcinoma (KIRP), LAML, brain lower grade glioma (LGG), liver hepatocellular carcinoma (LIHC), LUAD, LUSC, mesothelioma (MESO), ovarian serous cystadenocarcinoma (OV), pancreatic adenocarcinoma (PAAD), pheochromocytoma and paraganglioma (PCPG), prostate adenocarcinoma (PRAD), rectum adenocarcinoma (READ), sarcoma (SARC), SKCM, stomach adenocarcinoma (STAD), testicular germ cell tumor (TGCT), thyroid carcinoma (THCA), THYM, uterine corpus endometrial carcinoma (UCEC), uterine carcinosarcoma (UCS), and uveal melanoma (UVM).

The list of 7110 cancer somatic mutations in miRNA genes, together with sample identifiers and characteristics, was retrieved from Urbanek-Trzeciak *et al.* [table S2 in (*7*)]. To analyze the effect of mutations in miRNA genes on the generated miRNAs, we used sRNA-seq data of the corresponding TCGA samples mapped with the isoMiRmap tool (*36*) and retrieved from https://cm.jefferson.edu/isoMiRmap/ (*36*). IsoMiRmap allows precise annotation of miRNA length variants (isomiRs) and designated sequence variants, including miRNA reads with nontemplate nucleotides at the 3′ end and miRNA reads with mutations (substitutions and 1-nt indels) annotated in Catalogue of Somatic Mutations in Cancer (COSMIC) version 87 (released on 13 November 2018), covering a substantial fraction of the TCGA mutations (*7*). For each sample, we downloaded three separate files generated by isoMiRmap, i.e., “exclusive” (containing reads mapped to unique miRNA genes), “ambiguous” (containing reads mapped to multiple genome sites/miRNA genes), and “snps” (containing reads with genetic variants). A detailed description of isoMiRmap, the settings used for mapping, and the format of the output data can be found in (*36*).

To prevent any uncertainties, biases, or confounding factors, the following mutations were removed from the analysis: (i) mutations identified in redundant miRNA genes [e.g., *MIR1-1* (miR-1-1) and *MIR1-2*] (to avoid artifacts resulting from the ambiguous mapping of reads to gene copies), (ii) more than one mutation in a particular miRNA gene in one sample (such cases were very rare and not included in isoMiRmap), (iii) long indels (more than 4 nt) (long indels are rare, often wrongly annotated and not included in isoMiRmap), (iv) mutations in samples with no sRNA-seq data, (v) mutations in miRNA genes with one of the miRNA arms not defined in miRBase (such cases did not allow for an unambiguous distinction of miRNA gene subregions), (vi) mutations in samples with more than one sRNA-seq dataset (sequenced multiple times) (few cases; to avoid arbitrary assignment/selection of sRNA-seq datasets), (vii) mutations in miRNA genes for which no reads were mapped in the mutated samples (the lack of miRNA reads made it impossible to determine the effect of the mutations), (viii) mutations in samples with many ambiguous (mapping in multiple positions) reads in sRNA-seq (few mutations; to avoid determining the effect of mutations on the basis of low-quality data), and (ix) mutations located in mature miRNA or in proximity (±2 nt) to DROSHA/DICER1 cleavage sites but not annotated in COSMIC version 87 (mutations not annotated in COSMIC were not included in isoMiRmap].

On the basis of the analysis of the above data and the above-listed criteria, we created a dataset consisting of 1309 mutations (1218 unique mutations) (table S1). Table S1, in addition to data retrieved from (*7*) (yellowish columns), contains data generated in this study (white column). The data were generated with the in-house script prepared in R. The list of mutations collected in table S1 served as a base list for selecting mutations for subsequent experiments.

To analyze the effects of mutations on miRNA levels, isomiR distributions, strand balance, and the levels of miRNA targets (mRNAs) (analysis across multiple samples), we used crude sRNA-seq data retrieved from Loher *et al.* (*36*) and batch-corrected RNA-seq data retrieved from the supplementary file EBPlusPlusAdjustPANCAN_IlluminaHiSeq_RNASeqV2.geneExp.tsv from Hoadley *et al.* (*67*). The sRNA-seq data were batch corrected by us as previously described (*68*), considering all isoMiRmap-annotated isomiRs. We retained all isomiRs to ensure that those potentially unique to mutated samples were not excluded. Batch correction was performed separately for each of the 33 TCGA cancer types (cohorts), considering the following confounding factors: (i) the platform [Illumina Genome Analyzer (GA) or Illumina HiSeq], (ii) tumor purity, and (iii) plate (in which the cDNA library was prepared). We did not correct the data for the type of RNA isolation protocol [direct (total RNA) or MultiMACs (poly-A–depleted RNA)] because a single protocol was always used for a specific cancer type. Data about the platform and protocol were collected from the supplementary file from Hoadley *et al.* (*67*) and from the GDC Legacy Archive, tumor purity values were estimated using the TCGA tumor purity function from the TCGAbiolinks R package (*69*), and the plate IDs were identified on the basis of the aliquot barcodes. For batch correction, we used two algorithms: ComBat [version 3.80 from the sva R package (*70*)] or limma [version 3.6 from the limma R package (*71*) available as part of the Bioconductor project]. To identify potential confounders and select the proper algorithm, principal components analysis (PCA) was performed using the prcomp function of the stats R package (www.rdocumentation.org/packages/stats/versions/3.6.2/topics/prcomp). On the basis of the PCA results, the following statistical tests were performed: the Kruskal-Wallis test for categorical variables with *n* > 2, the Wilcoxon rank-sum test for categorical variables with *n* = 2, and the Kendall tau rank correlation for ordinal variable purity. The effectiveness of the performed batch corrections was estimated using the false discovery rate *P* value, which indicates the association between potential confounders and the principal component, and visualized with the PCA and t-distributed stochastic neighbor embedding graphs (Rtsne wrapper for the Barnes-Hut t-Distributed Stochastic Neighbor Embedding from the Rtnse R package was used). On the basis of the above, limma was chosen for all tumor cohorts that required correction [CHOL, glioblastoma (GBM), and UVM did not show any batch effects and thus were not batch corrected].

### miRNA precursor structure modeling

The secondary structures of the wild-type and mutant miRNA precursors and the change in Gibbs free energy (revised/optimized dG value) of the structures and individual base pairs or regions were predicted with the use of mfold version 3.6 or the mfold web server (*72*) with default parameters and processed in VARNA (*73*). For modeling, we used pre-miRNA sequences [reconstructed on the basis of miRBase as in (*7*, *74*)] extended upstream (5′) and downstream (3′) by 25-nt flanking sequences. The structures with the lowest free energy are presented in this study. Spatial (3D) structures were modeled (on the basis of the predicted secondary structures) using RNAComposer software with the default parameters (*75*) and visualized via PyMOL (*76*).

### miRNA level analysis

Analysis of the influence of miRNA gene mutations on the miRNA level was performed using two different approaches. In the first approach (Experiment_1), within a specific mutant sample, we directly compared the levels (number of reads) of miRNA from the wild-type and mutant alleles. To evaluate the FC (or depletion) of the mutated allele, the numbers of reads at the RNA level were subsequently compared with the corresponding numbers of reads of mutant and wild-type alleles at the DNA level, and the significance of the difference was calculated with Fisher’s exact test. This approach was applied only for mutations in mature miRNA sequences that can be distinguished at the RNA level. For the analysis, we considered only the “exclusive” and “mutated” types of reads (wild-type and mutant). To select mutations adequate for the test and to avoid any uncertainties, the following mutations were excluded from the analysis: (i) not located in mature miRNA and (ii) with fewer than 50 total miRNA reads in a mutated sample (not distinguishing reads from wild-type and mutant alleles). In the second approach (Experiment_3), in which we analyzed mutations located in all miRNA gene subregions (not only in mature miRNAs), we compared the level of miRNA in a mutated sample with the levels of miRNA in other samples of the same cancer type (without the mutation). For the analysis, we used batch-corrected reads per million (RPM) values. The level of a specific miRNA gene in a specific sample was calculated as the total RPM of all 5p and 3p isomiRs derived from a particular gene, including “exclusive,” “nontemplate,” and “mutated” reads. To increase the normality of the miRNA level distribution, the RPM values were log_2_ transformed (RPM + 1). To avoid uncertainties, mutations with an RPM value lower than 10, either in a mutated sample or the average RPM of the other samples, were excluded. The level of a miRNA in a sample with a mutation versus that in samples without mutations was compared with a one-sample *t* test and *z*-score. To minimize the chance of false-positive results, only mutations with (i) a *t* test with a Bonferroni-corrected *P* < 0.05, (ii) a *z*-score *P* < 0.05, and (iii) |log_2_FC| > 1 and those (iv) identified as outliers defined as a data point >1.5 IQR (interquartile range) below Q1 or above Q3 were considered significant.

### IsomiR classes

Consistent with the convention described previously (*38*), all isomiRs were categorized into nine basic classes denoted as follows: 0|0 [reference (canonical) isomiR as annotated in miRBase]; 0|−; 0|+; +|0; −|0; +|−; −|+; +|+; −|−. The signs/values before and after the vertical line correspond to the 5p and 3p miRNA ends (5p|3p) and indicate the direction of the particular end shift, i.e., no change (0), upstream [3p to 5p, (−)], and downstream (+). In addition, when comparing isomiR profiles between samples (mutant versus wild type), reads with nontemplate 3p end modifications were considered an additional class of (nontemplate) isomiRs, denoted (nt) if any modification was present. The extension of the classification may be used to indicate the exact isomiR coordinates, e.g., −2|−1 (indicating an isomiR with the 5p end shifted by 2 nt upstream and the 3p end shifted by 1 nt upstream) or by using (*n*) for any nucleotide change at the 3p end to emphasize the modifications at the 5p end. The principles of isomiR classification and denotation are shown in fig. S2.

### IsomiR distribution analysis

As in the case of the analysis of the effect of mutations on the level of miRNA, the effect of mutations on isomiR distribution was analyzed using two different approaches. In the first approach, we compared the fraction of isomiRs (isomiR profiles) of mutant and wild-type alleles, which were divided into nine isomiR classes (as defined above), within one sample. To calculate the number and fraction of reads classified into particular isomiR classes, we used raw read counts (only the “exclusive” and “mutated” types of reads were taken into account). Rare cases of isomiRs/reads for which it could not be determined whether they originated from wild-type or mutant alleles were excluded from the analysis. From the list of mutations (table S1), we excluded mutations that (i) are not located in mature miRNAs and (ii) have <50 reads derived from either allele. For the statistical analysis of changes in isomiR profiles, we used Pearson’s chi-square independence test and Cramer’s *V* test. For the chi-square test, we added a pseudocount (number of reads +5) to each isomiR class. Cramer’s *V* value measures the strength of the association/relationship between two variables. Cramer’s *V* values of <0.1, 0.1 to 0.2, 0.2 to 0.4, and >0.4 were interpreted as no association or small (weak), medium, and large (strong) associations, respectively (*77*).

In the second approach, we compared the isomiRs (isomiR profiles) derived from a mutated gene in the sample with the mutation and in other samples of the same cancer type without the mutation. From the list of mutations (table S1), we excluded mutations that had less than 20 RPM for a particular miRNA strand in the mutated sample and less than 20 RPM for the average in the corresponding wild-type samples. If the criteria were fulfilled for both strands, the isomiR profiles were analyzed for each strand. As described in the subchapter above, isomiRs were divided into 10 classes, and fractions of particular isomiR classes were calculated on the basis of batch-corrected RPM values. For each miRNA of interest, we subsequently calculated the distance (sum of differences of all isomiR fractions; values from 0 to 2 where 0 indicates no difference and 2 indicates the occurrence of completely different isomiRs) between the isomiR profile in a sample with mutation and an average isomiR profile of samples without mutation. The distance calculated for the sample with the mutation was compared (one-sample *t* test and *z*-score) with similarly calculated distances of all samples without the mutation and visualized on a cumulative graph of isomiR profiles. Only mutations with (i) a *t* test with a Bonferroni-corrected *P* < 0.05, (ii) a *z*-score *P* < 0.05, and (iii) a distance from the average isomiR profile of samples without a mutation >0.2 were considered significant.

### Strand balance analysis

Strand balance analysis was performed by comparing the 5p/3p strand balance [calculated as log_2_(5p_raw_counts+1/3p_raw_counts+1), including “exclusive,” “nontemplate,” and “mutated” reads] of a mutated miRNA gene in a mutated sample versus the strand balance of the gene in other samples of the same cancer type without mutation. To select mutations that were adequate for the test and to avoid any uncertainties, mutations with fewer than 50 total reads for a particular miRNA gene (table S1) were excluded from the analysis. To ensure a reliable estimation of the strand balance in the other (reference) samples, only samples with a total number of reads ≥50 were included in the analysis. To identify mutations that significantly affect strand balance, the values were compared with a one-sample *t* test and *z*-score. Only mutations with (i) a *t* test with a Bonferroni-corrected *P* < 0.05, (ii) a *z*-score *P* < 0.05, and (iii) >10% change in strand fractions between the mutated sample and the average from other wild-type samples and those (iv) identified as outliers, defined as a data point >1.5 IQR below Q1 or above Q3, were considered significant.

### Differential expression

For differential expression analysis, we used batch-corrected mRNA (RNA-seq) data. The analysis was performed with the use of the DESeq2-based (*78*) R pipeline (SARTools) (*79*) to compare selected samples with mutations affecting the analyzed aspects of miRNA gene biogenesis with other samples of the same cancer type without a mutation.

### miRNA target prediction

miRNA target prediction was performed using TargetScan (*80*) and mirDB (*81*), allowing target prediction for both wild-type and mutant (custom) sequences or isomiRs. For the prediction of mutation-specific targets, we used the most common and/or most differentiated isomiRs in a mutated sample. We subsequently compared the list of predicted targets with the list of differentially expressed genes between the wild-type and mutant samples.

### Experimental analysis of the effect of mutations in *MIR142* and *MIR205*

The human embryonic kidney cell line HEK293T (no. CRL-3216, American Type Culture Collection, Manassas, VA) was cultured in high-glucose Dulbecco’s modified Eagle’s medium (no. 11960044, Gibco, Carlsbad, CA) supplemented with 10% fetal bovine serum (no. 10500064, Gibco), 1% GlutaMAX (no. 35050038, Gibco), and 1% antibiotic-antimycotic (no. 15240062, Gibco). Cells were transfected with plasmids pCDH-CMV-MCS-EF1α-GreenPuro (no. CD513B-1, SBI, Palo Alto, CA) encoding wild-type and mutant precursors of *MIR142* and *MIR205*, defined as sequences encoding pre-miRNAs (on the basis of miRBase) extended by immediately adjacent 25-nt flanking sequences. The *MIR142* mutants include n.55A>G, n.59C>T, and n.85G>A, and the *MIR205* mutant includes n.97_98dupGG. For transfection, Lipofectamine 2000 (no. 11668030, Invitrogen, Waltham, MA) was used.

For sRNA-seq, only *MIR142* mutants were used. Twenty-four hours after transfection, total RNA was isolated from the cells using TRIzol (no. 15596026, Invitrogen). Library preparation and sRNA-seq were conducted by the Novogene Company using the NEB Next Multiplex Small RNA Library Prep Set for Illumina and the Illumina HiSeq 6000 system in single-end 50–base pair mode. Raw sequencing reads in FASTQ format were processed to remove adaptors and subsequently filtered for quality. Reads ranging from 16 to 30 base pairs in length were extracted and mapped to reference miRNA precursors (miRBase) using Bowtie (*82*).

Mature miRNA isomiRs (5p or 3p) were defined as variants whose 5′ ends shift up to 10 nt upstream or downstream relative to the predominant sequence. For each arm, isomiRs were categorized into nine classes, as described before. Expression levels were normalized by dividing each isomiR read count by the reads of an internal reference miRNA [misafe (*83*), designed to not target human genes, which was also cloned to the expression plasmids]. Experiments were performed in triplicate or in duplicate for n.85G>A.

To evaluate the impact of mutations on the silencing efficiency of miRNA targets, we used a dual-luciferase reporter assay. Using Lipofectamine 2000, we cotransfected cells with either wild-type or mutant expression vectors, along with plasmids pmirGLO (no. E1330, Promega, Madison, WI) containing sequences of a perfect-match or naturally occurring validated targets of miR-142-5p and miR-142-3p (*17*) and perfect-match or natural targets selected on the basis of the complementarity and number of miRNA binding sites using TargetScan of miR-205-5p and miR-205-3p (figs. S7 and S8), inserted into the 3′ untranslated region of the luciferase gene. Luciferase activity was measured 24 hours after the transfection using the dual-luciferase reporter assay (no. E1910, Promega) following the manufacturer’s protocol. All experiments were performed in triplicate. The efficiency of target silencing by particular miRNAs was calculated in reference to cells cotransfected with pmirGLO with a particular target sequence and empty (without tested miRNA) pCDH-CMV-MCS-EF1α-GreenPuro [negative control (NC)].

### Statistical analysis

Statistical analyses were performed using the stats R package, GraphPad Prism, or the Real Statistics Resource Pack for MS Excel (https://real-statistics.com/). Particular statistical methods are described in the previous sections of Materials and Methods corresponding to each separate experiment and analysis.
