# Supplementary material for: miRNA gene mutations commonly disrupt the proper functioning of miRNA genes
Source: Sci Adv. 2026 Feb 18;12(8):eaea6079. doi: 10.1126/sciadv.aea6079 (PMC12915624; doi:10.1126/sciadv.aea6079)
Supplement: Supplementary file 1 — Figs. S1 to S9 Legends for tables S1 to S8 [file sciadv.aea6079_sm.pdf]

Science Advances
AAAS

Supplementary Materials for

miRNA gene mutations commonly disrupt the proper functioning of miRNA genes

Magdalena Machowska *et al.*

Corresponding author: Piotr Kozlowski, kozlowp@ibch.poznan.pl

Sci. Adv. **12**, eaea6079 (2026)
DOI: 10.1126/sciadv.aea6079

The PDF file includes:

Figs. S1 to S9
Legends for tables S1 to S8

Other Supplementary Material for this manuscript includes the following:

Tables S1 to S8

Supplementary Figures

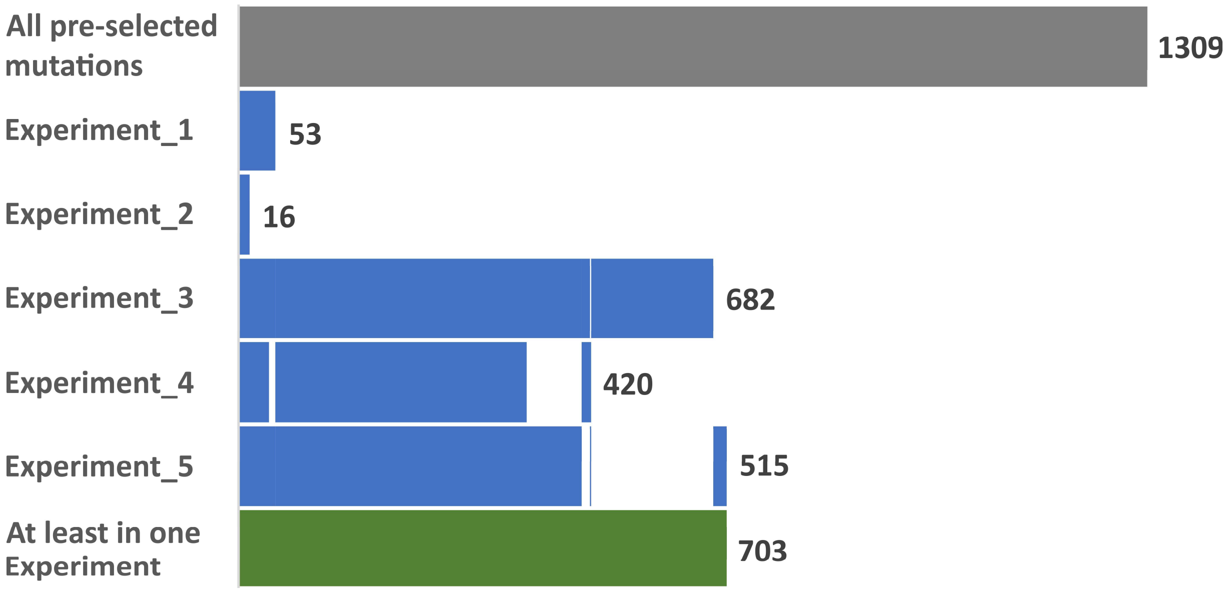

Supplementary Figure S1. The number and overlap of mutations analyzed in the study. *The gray, blue and green bars indicate all mutations preselected for the study, mutations selected for subsequent Experiments and mutations used in at least one Experiment, respectively. The blue and green bars are juxtaposed with the order of mutations in the gray bar, which are sorted according to their selection for the subsequent Experiments.*

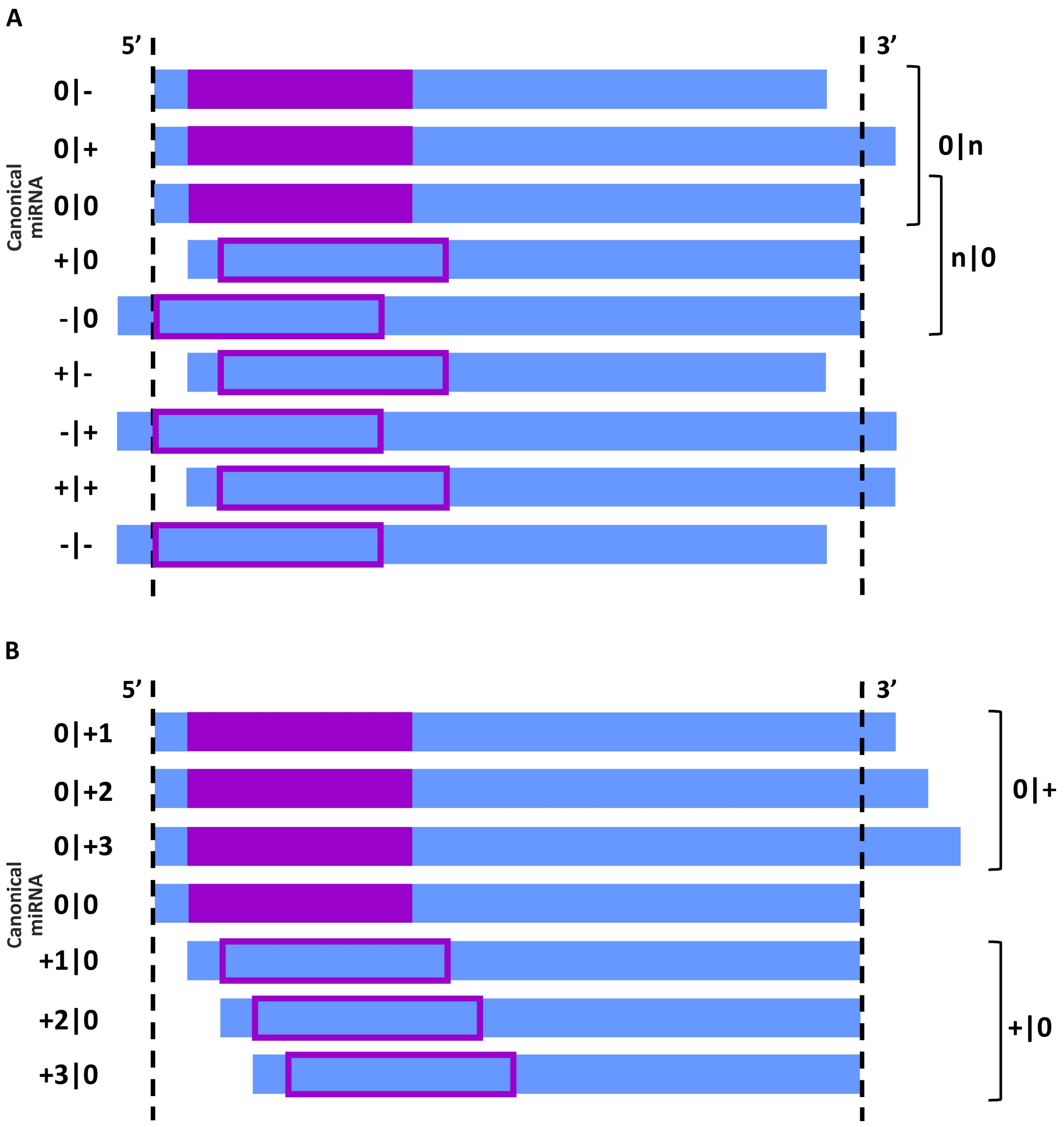

Supplementary Figure S2. Principles of the classification and denotation of isomiRs. *The canonical (0|0) and different classes of isomiRs are shown as light blue rectangles; the canonical seed sequence is marked as a purple rectangle, and altered shifted seeds are marked as open rectangles. A – Principles of isomiRs denotation on the basis of the direction of the end shift; ‘n’ denotes any shift at a given end of the mature miRNA. B – Principles of denotation of the size (nucleotide number) of the end shift.*

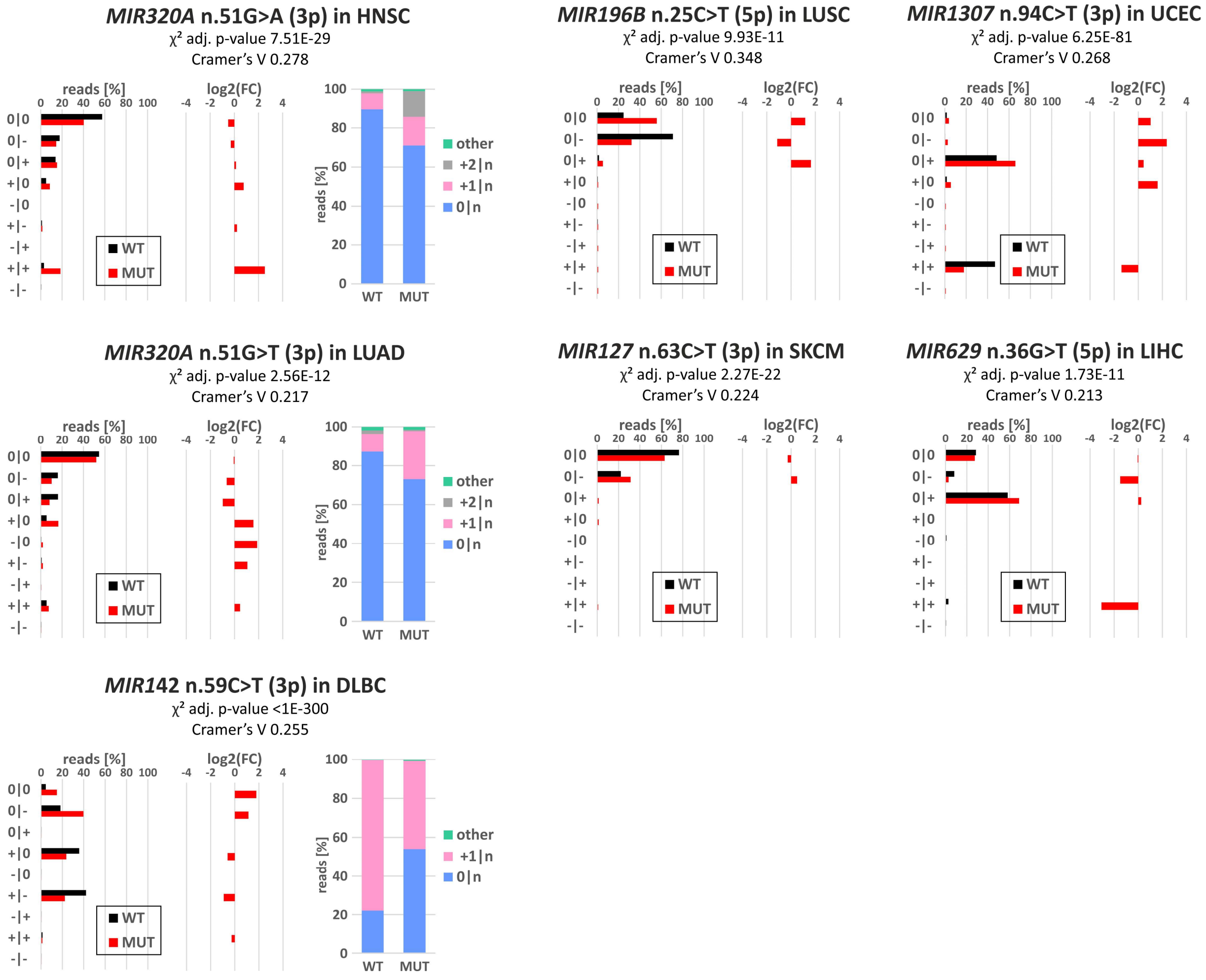

Supplementary Figure S3. Effects of miRNA gene mutations on isomiR profiles (Experiment_2). *The figure scheme as in Figure 3D and F.*

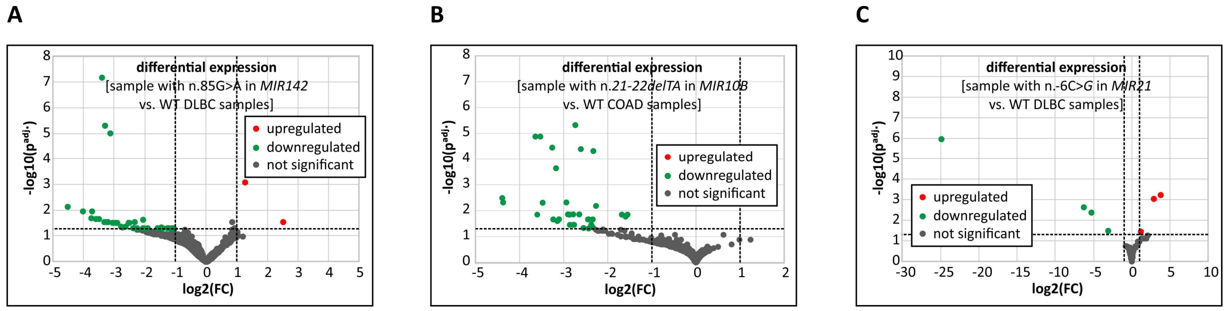

Supplementary Figure S4. Differential expression analysis of samples with mutations affecting isomiR profiles (Experiment_4). *A – n.85G>A in MIR142, B – n.21-22delTA in MIR10B, C – n.-6C>G in MIR21, vs. corresponding WT samples. IsomiR profiles for these mutations are presented in Figure 5 C, D and E, respectively. Red, green, and gray dots indicate upregulated, downregulated, and not significantly changed genes, respectively.*

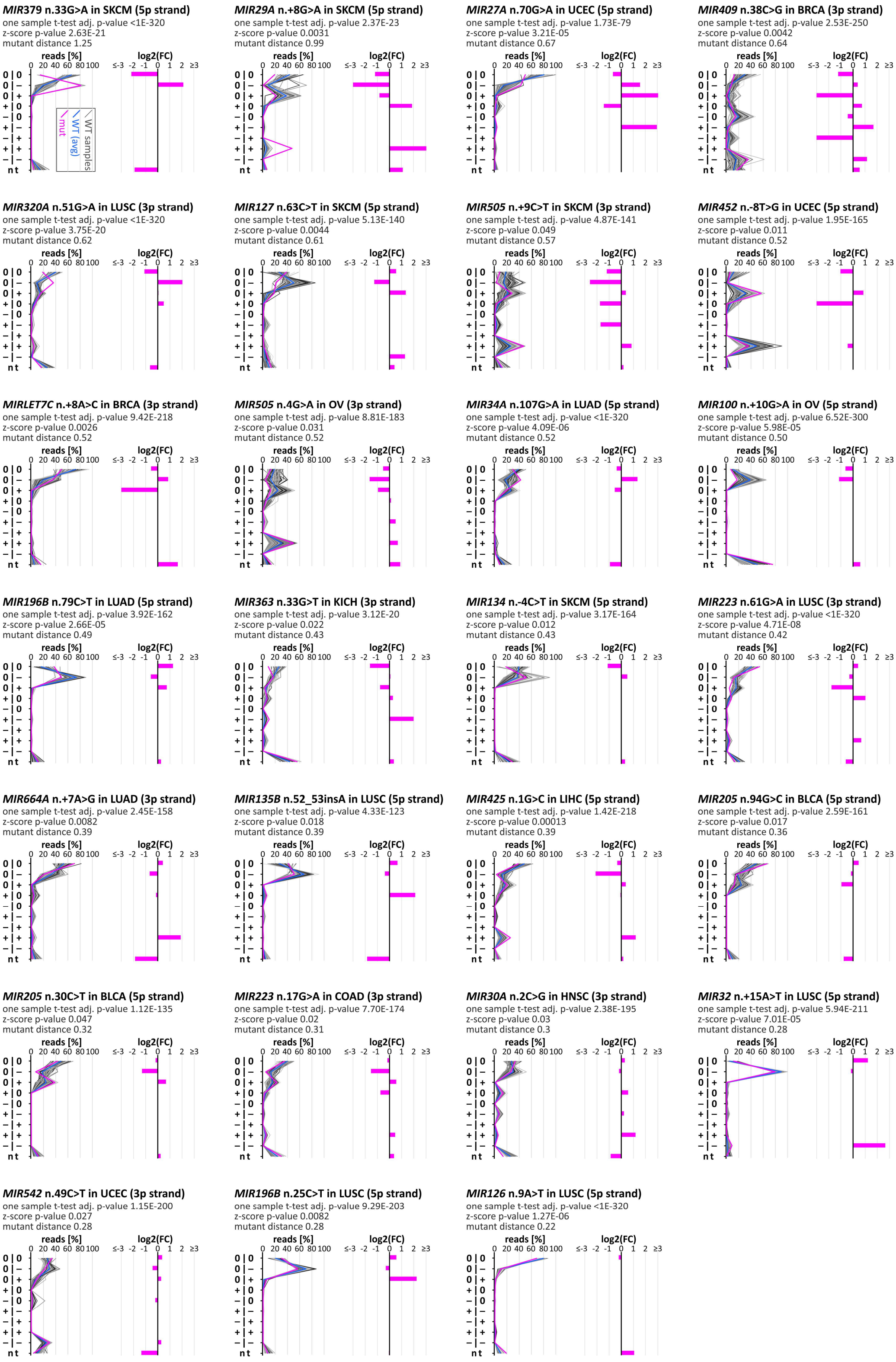

Supplementary Figure S5. Effects of miRNA gene mutations on isomiR profiles (Experiment_4). *The graph scheme as in Figure 5C, D and E.*

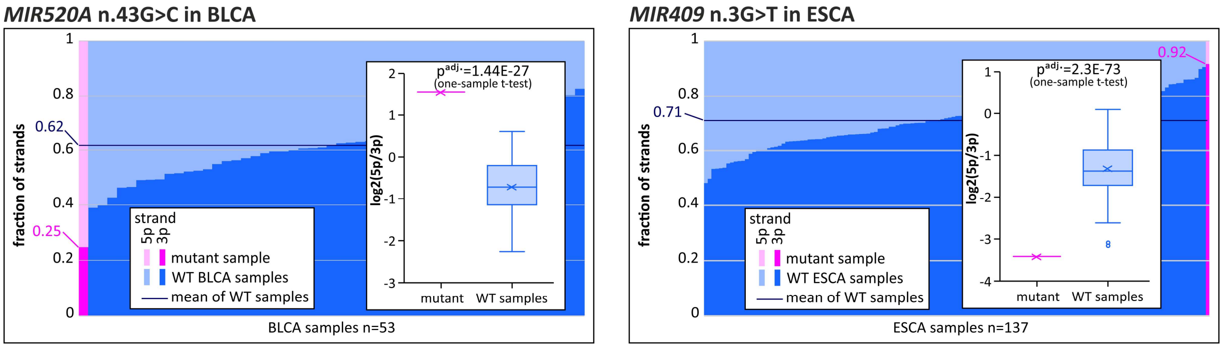

Supplementary Figure S6*. *Effects of miRNA gene mutations on the balance of miRNA strands (Experiment_5). *Influence of n.43G>C in the loop of MIR520A and n.3G>T in the 5p-flank of MIR409 on the change in the balance of miRNA strands. The graph scheme as in Figure 6C and D.*

Natural *MIR142* targets

TGFBR1

AGCTGTGTTTCCCCATAAGTTTTTTTAAATTGTATATTGTATTTGTAGTAATATTCCAAAAGAATGTAAATAGGAAATAGAAGAGTGATGCT
TATGTTAAGTCCTAACACTACAGTAGAAGAATGGAAGCAGTGCAAATAAATTACATTTTTCCCAAGTGCCAGTGGCATATTTTAAAATAAAG
TGTATACGTTGGAATGAGTCATGC

Identified target sequence:
mir-142-3p target sequence

RAC1

CAGAACTGCTATTTCCTCTAATGAAGAATTCTGTTTAGCTGTGGGTGTGCCGGGTGGGGTGTGTGTGATCAAAGGACAAAGACAGTATTTTG
ACAA**AATACGAAGTGGAGATTTACACTACA**TTGTACAAGGAATGAAAGTGTCACGGGTAAAAACTCTAAAAGGTTAATTTCTGTCAAATGCA
GTAGATGATGAAAGAAAGGTTGGTATTATCAGGA

Identified target sequence:
mir-142-3p target sequence

ASH1L

CATCCTCAGATCCCACAGGCATTGCGAAGAAGCTGTGCAAATGGAGATTCAGTTCAAAATAAAATCATTTAAAAACCTACATAAAAAGAACT
CTAAACCCA**CCCCTGCAACAAAAGTCACTACA**TAAACTGTTCAGCAGTATTCACCTATCAGAGTATTTGTTGTGAGTATA**GATTATCAATTG**
AAAACACTACTCTTGTTTTCTTAATTGTACAGTTTTCAATGTCCCTTTCTTAAAGAGACAGTATATTTCTCTTCACCCCTAGCCCATCTTCC
CTCACCCTCCTGAATGACATCAGGAGGTATATCCAGGGTGTCTCCTTCCTTCCTACTCTCTTGACCAGAAGTTAACAGACTATACTGTCTCT
TTAAAAATAAAATTTAAAAAGCTTTGTTGTCTTTTCAGACATACATATGCATATATGTTTTAGATGTTCTTATAAGAGAAAAGATGGTTTTT
AAATGTGCCAAGTTGTGTGTGTGTGTGTATATATATGTGTGTATGTGTGTGTATATATATATGTGTGTGTGTATATATATACACACACACAC
ACACACCTGCTGTGTGATTGGTAAGCAATACAATAGTAAACATGTCCCCATTACTTTTTTCTAATATTGGACCAATGCTGTCCTAATTGTAC
ATTTCCCCTTATGGTGACGATGCTCTGACTCGTTTAGGTAGACACATTGACCACCTTCCATTCCATTAAATATTTTTTCCTTTTTCCCCTTT
CTGTGTCATTCTTGAGGAAAAAACAAAAGAGAGAGGGGATGCCAATGATCCCCTTGAGCAGAGAAAAAGCAAAATAAATATTTTATTAAAGA
AAAAAGAGAATTAAGAAAATAGTTTGGAGTATTTTCTTACTGTAGAGAAGCACTGTACATTACTAAGAGACCTGGGTATAAGATACTCACAT
GTGGAGCTGGAAAAATCGCATGTCCAAGCCCGTTTGAGTGGTTTCTTTTGTTTTTCATTGCAGGGAGTGGGTGGGAGGGAGGTGGGACTAGG
GGCACTTTGGGGGTCTCCTTTTAGTCAAAAGCGAGAAAATGACAAGAAAGAGATTA**AAATTCAATGTTTCCTTTATA**GTGTTAAACACTAAA
ATTTTAAAAAAGATGAAAAAGAAAAAAAAACTTTGTAAAATGCGA**GAACAGAAGCAAAAGACACTACG**CTCTGTCATTTTATCTTTCTTTTG
TTGAAAGACTAAAAAAAAACTGAAATGTTTTTTAGACAATCAAATGTTAGGTAAGTGCAAAAACTTGTTTTTTCTTACTGGTGTAGAAATTA
ATGCCTTTTTTTATTTTTCAGTTATTTTATAATAACGAAATAAAAAGAACCCCCCAGCTGCCAGGCGGGTTTTGGTGTTT**GAAATGCGGGGC**
AAAGCACTACATCACTGCAAATAGATACAGAGTTAGTCTGCATGTCTGTAGGCTGTGTGATTGCG

Identified target sequences:
mir-142-3p target sequence 1
mir-142-3p target sequence 2
mir-142-3p target sequence 3
mir-142-3p target sequence 4
mir-142-5p target sequence 1

Natural *MIR205* targets

CHN1

TTTTTAATTTGAGGGGAAAA**GAAATGTTTTACAGATGAAGGA**ATGTTTTATAGTAATTTAATTTGCTCCTGTAGCTGCATTATTTCTTGATT
AGAGGTTTGGGCAT**ATAACCAGATTAAAGTGAAGGA**ACTTTCTGTTGTTTTT]GTAGCACCGCTCAGCTGTCTTGTAAAACAGTGAACACAC
GCTTTCTGGTTCTAGTAATCCTGGG

Identified target sequences:
mir-205-5p target sequence 1
mir-205-5p target sequence 2

MPP16

AGCCAGTGGTCATTAGATGTTACTGTGCTTAAACACATCTGATACTCATGCATTGCCTTACAGGCTAAAAATGTAAGAGGTAGGTGTTCAGT
AACCTCAAGTTGGAAAGATATATAATGTTAGAATCCCCTGAG**ACAAGCCATTTCCCTGAAATA**GAAATTTACCTCAATACTCATGTAATCTA
AGACACTTGGCGTAGTAGCAAAGAAGTTGCCTTCATGTTCTAGTCTCATCTAATTGTGACCTTT**GTCTTTCATGTACTTCTGAAATC**ATTTT
CCACTTTTTCCACTGGTGTGA**TTTTGAAACAATTCTGAAATA**TTTCAGGTAAGATTAATAACATCCAATTACAAATATATGTTTCAATATTT
TATACGTATGTCTACTTTGAAAGTTAAACCAATAGTATAGAAAGCCTAAGAATGAACACTGATTGGACATACTCACAGAAATTAAGGGAAAA
ACACATATTGTAAAATTCCTGTCAATGTTTGAGTAGAATACAGAAGTACATAGCAGTCTTCAATTTTTAAACACAATTATGGGCTTATAACT
GGACGTGAC

Identified target sequences:
mir-205-3p target sequence 1
mir-205-3p target sequence 2
mir-205-3p target sequence 3

Supplementary Figure S7. Target sequences of miR-142-5p/3p and miR-205-5p/3p used in functional experiments. *Sequences were inserted into the 3'UTR of the luciferase gene in the vector used in the dual-luciferase assay. Sequences that directly interact with specific miRNAs are highlighted in color frames; cloning primer sequences are underlined.*

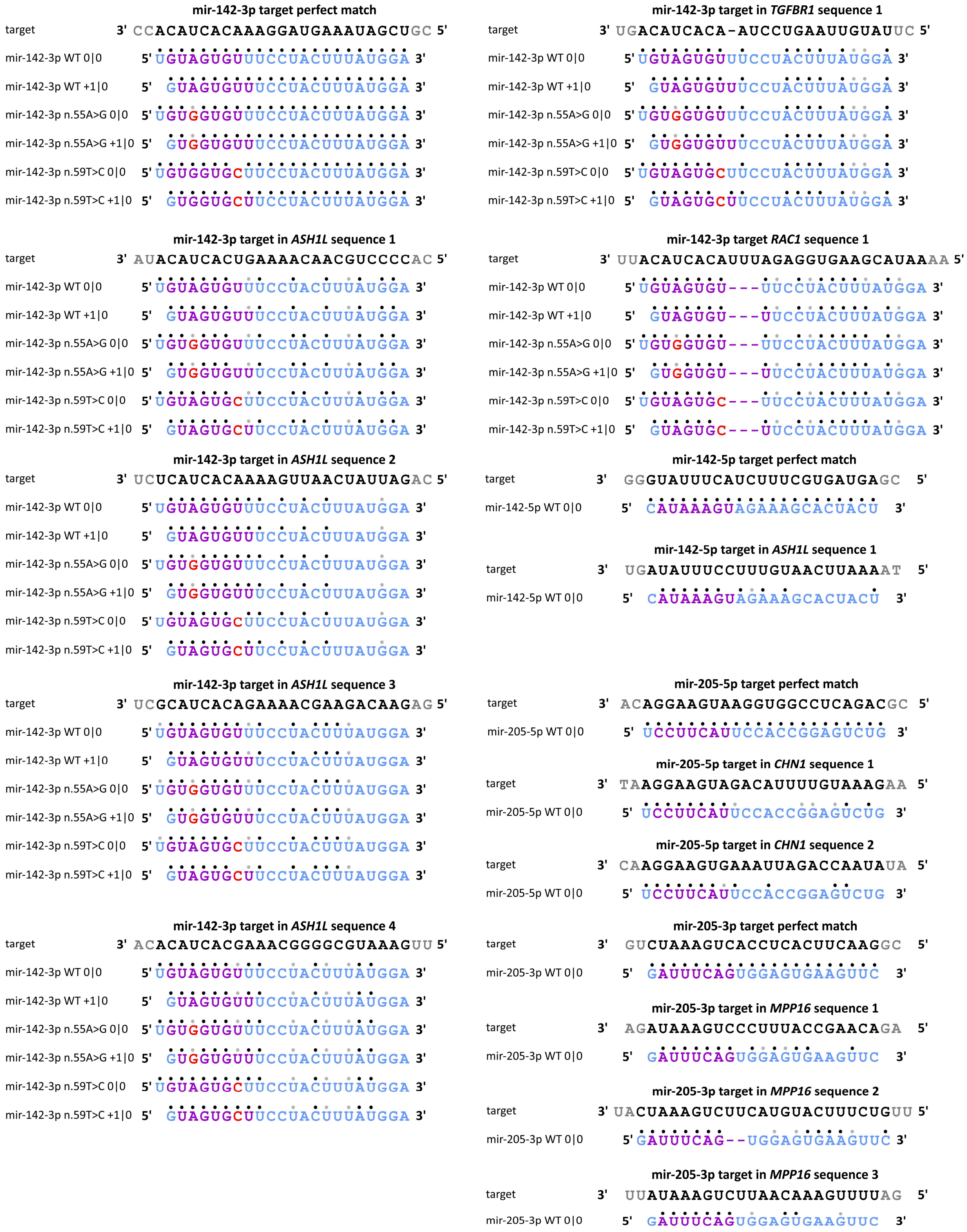

Supplementary Figure S8. Complementarity of WT and mutant miR-142-5p/3p and miR-205-5p/3p to their target sequences used in the dual-luciferase assay. *Black and gray dots represent Watson-Crick and Wobble pairings, respectively.*

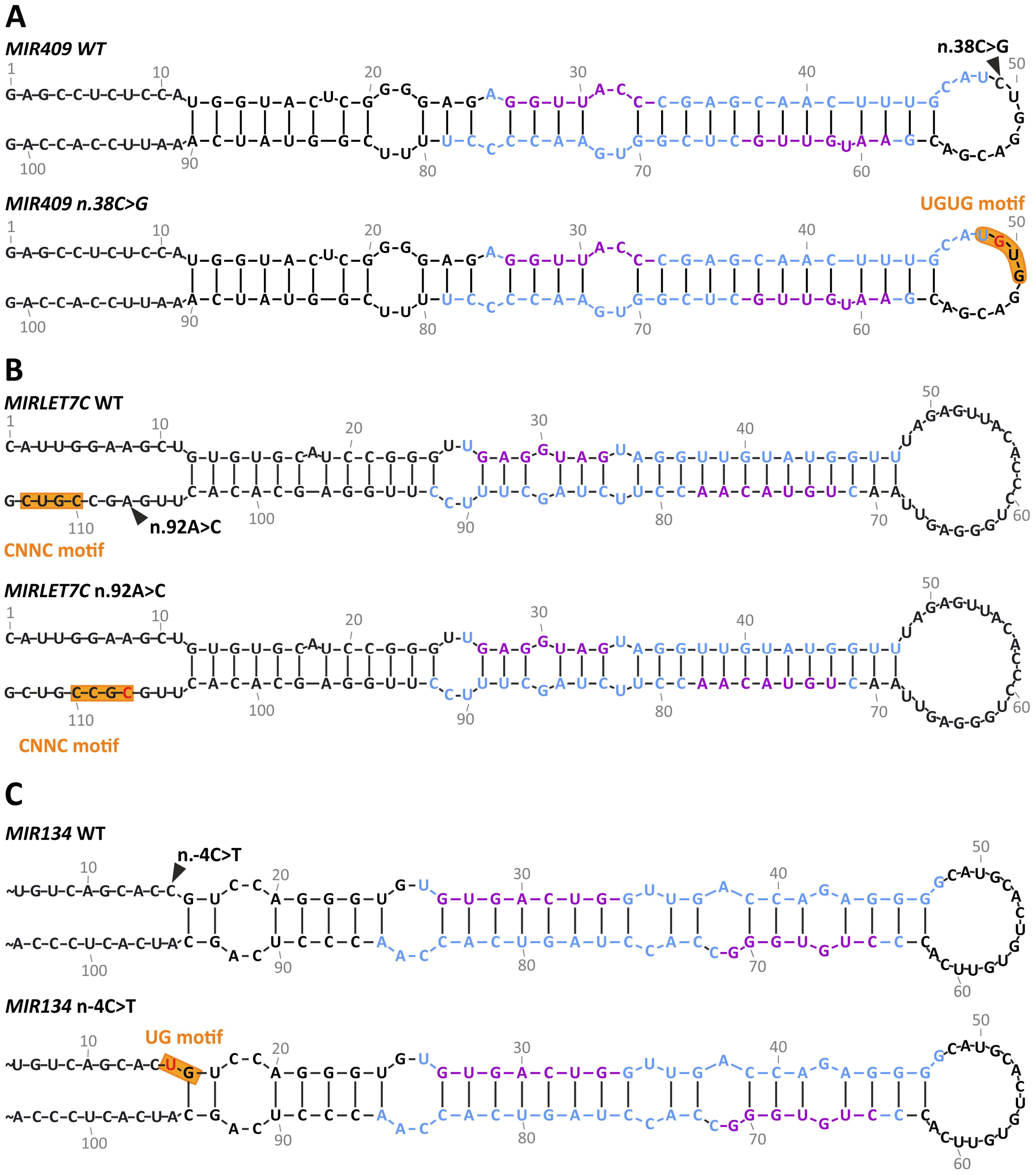

Supplementary Figure S9. Examples of mutations affecting miRNA-processing regulatory motifs. *A – mutation n.38C>G in MIR409 creates a new UGUG motif in the loop. B – mutation n.92A>C in MIRLET7C creates a new CNNC motif in the 3' flanking region. C – mutation n.-4C>T in MIR134 creates a new UG motif in the 5' flanking region at the basal junction.*

Supplementary Table S1 (separate file)
The list of mutations preselected for the analysis.

Supplementary Table S2 (separate file)
The list of mutations selected for Experiment_1 (green and red font indicate mutations significantly downregulating or upregulating miRNA levels, respectively; analysis was performed between WT and MUT alleles within one sample).

Supplementary Table S3 (separate file)
The list of mutations selected for Experiment_2 (red font indicates mutations significantly affecting isomiR profiles; analysis was performed between WT and MUT alleles within one sample).

Supplementary Table S4 (separate file)
The list of mutations selected for Experiment_3 (green and red font indicate mutations that significantly downregulate or upregulate miRNA levels, respectively; analysis performed between the MUT sample and WT samples).

Supplementary Table S5 (separate file)
The lists of differentially expressed genes in samples with mutations: n.5-7delAGC in *MIR122* and n.85G>A in *MIR518E* (Experiment_3, Figure 4E and F); n.85G>A in *MIR142*, n.21-22delTA in *MIR10B* and n.-6C>G in *MIR21* (Experiment_4, Supplementary Figure S4 A,B,C); n.97-98dupGG in *MIR205* and n.84C>T in *MIR365B* (Experiment_5, Figure 6E and F); green and red font indicate significantly downregulated or upregulated genes, respectively.

Supplementary Table S6 (separate file)
The list of mutations selected for Experiment_4 (red font indicates mutations significantly affecting isomiR profiles; analysis performed between the MUT sample and WT samples).

Supplementary Table S7 (separate file)
The list of mutations selected for Experiment_5 (red font indicates mutations significantly affecting miRNA strand balance; analysis performed between the MUT sample and WT samples).

Supplementary Table S8 (separate file)
Influence of mutations on the stability (free energy) of pri-miRNAs (blue font indicates mutations significantly affecting miRNA biogenesis in at least one Experiment).
